# Palladium-Catalyzed Sulfinylation of Aryl- and Alkenylborons with Sulfinate Esters

**DOI:** 10.1021/acs.orglett.1c01292

**Published:** 2021-04-28

**Authors:** Minori Suzuki, Kazuya Kanemoto, Yu Nakamura, Takamitsu Hosoya, Suguru Yoshida

**Affiliations:** † Laboratory of Chemical Bioscience, Institute of Biomaterials and Bioengineering, Tokyo Medical and Dental University (TMDU), 2-3-10 Kanda-Surugadai, Chiyoda-ku, Tokyo 101-0062, Japan; ‡ Department of Biological Science and Technology, Faculty of Advanced Engineering, Tokyo University of Science, 6-3-1 Niijuku, Katsushika-ku, Tokyo, 125-8585, Japan

## Abstract

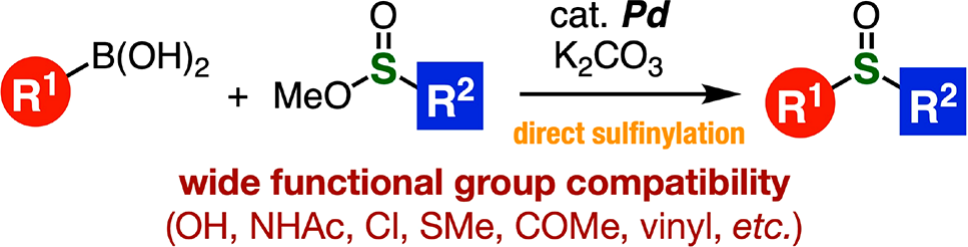

An efficient, direct sulfinylation of organoborons catalyzed by palladium is disclosed. Treatment of organoborons and sulfinate esters in the presence of a palladium precatalyst provided a broad range of sulfoxides. Various organosulfur compounds having oxidizable functional groups were successfully prepared through the sulfoxide synthesis.

Sulfoxides are a fundamental class of compounds in a broad range of research fields such as synthetic organic chemistry, pharmaceutical sciences, agrochemistry, and materials chemistry.^1,2^ Particularly, recent remarkable progress on versatile transformations of sulfoxides have allowed us to synthesize a wide variety of molecules.^2^ These recent advances clearly enhanced the synthetic utility of not only chiral but also achiral sulfoxides.^2^ Despite their great significance, accessible sulfoxides by conventional methods through sulfanylation of Grignard reagents, organic bromides, or organoborons and following *S*-oxidation are limited since various functional groups can be damaged in the oxidation step (Figure 1A).^2,3^ Thus, an efficient method for direct sulfinylation is highly sought after. We herein describe a direct method for sulfinylation of aryl- and alkenylborons with sulfinate esters catalyzed by palladium, enabling the preparation of a wide variety of sulfoxides having easily oxidizable functional groups.

**Figure 1:**
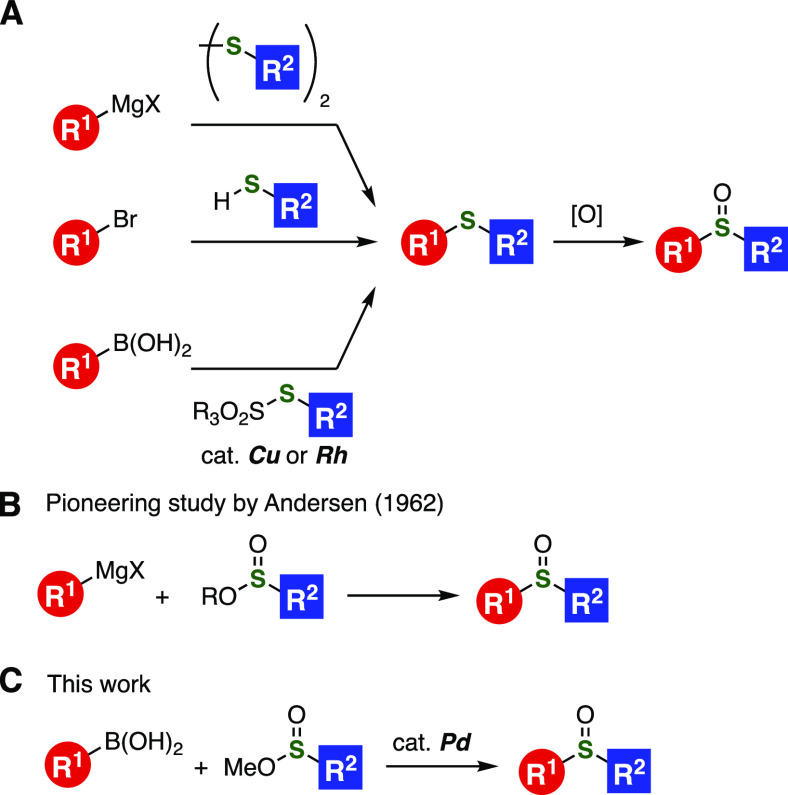
(A) Conventional methods for sulfoxide synthesis. (B) Pioneering study. (C) This work.

Conventional direct sulfoxide synthesis has been achieved from nucleophilic carbanions with sulfinate esters as a sulfur surrogate.^4−6^ A pioneering study on the sulfinylation of organomagnesiums using sulfinate esters was reported by Andersen and co-workers in 1962 (Figure 1B).^4^ Considering that recent significant successes of modern organometallic chemistry have greatly improved the availability of diverse molecules including biaryls and amines, an efficient cross-coupling reaction using sulfinate esters is highly demanded for synthesizing diverse sulfoxides. With our recent achievements in organosulfur chemistry using thiosulfonates catalyzed by transition-metals in mind (Figure 1A, bottom),^7^ we envisioned that a wide range of sulfoxides can be prepared by direct sulfinylation of organoborons catalyzed by a transition-metal complex using sulfinate esters as electrophilic sulfur surrogates under mild conditions (Figure 1C).

A sulfoxide synthesis from 4-tolylboronic acid (**1a**) and methyl 4-methoxybenzenesulfinate (**2a**) was chosen as a model reaction (Table 1). After a number of examinations, we found that a catalytic amount of Pd(dba)_2_ with XPhos as a ligand promoted the sulfinylation in the presence of potassium carbonate in 1,4-dioxane and water (v/v = 10/1) at 80 °C (entry 1). In contrast, the yields of sulfoxide **3a** were significantly decreased when the reaction was conducted using triphenylphosphine or an *N*-hetero cyclic carbene ligand (entries 2 and 3). Palladium precatalysts XPhos Pd G3 and XPhos Pd G4 also catalyzed the synthesis of sulfoxide **3a** (entries 4 and 5).^8^ While the reaction performed at 100 °C lowered the efficiency (entry 6), the yield of **3a** based on recovered starting material was improved by decreasing the reaction temperature to 40 °C (entry 7). Although the reaction without water decreased the yield of **3a** (entry 8), we accomplished the synthesis of sulfoxide **3a** in high yield by changing the ratio of solvents from 10/1 to 5/1 (entry 9). Sulfoxide **3a** was obtained in low yield when further increasing the ratio of water (entry 10). We succeeded in decreasing the catalyst loading from 10 to 5 mol % (entry 11). Further improvement of the efficiency was achieved by increasing the amount of **1a** and decreasing the concentration of substrates from 0.1 to 0.05 M, enabling us to prepare sulfoxide **3a** in high yield (entry 12). The reaction with a catalytic amount of XPhos in the absence of palladium precatalysts did not afford sulfoxide **3a**, in which 94% of sulfinate ester **2a** was recovered. This result clearly showed that palladium catalyzed the sulfinylation of 4-tolylboronic acid (**1a**) with sulfinate ester **2a**.

**Table 1:**
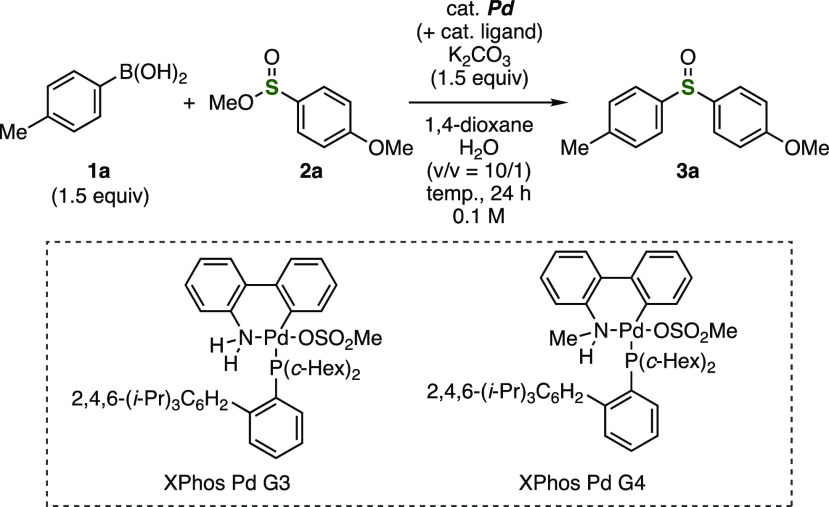
Optimization of the Reaction Conditions

entry	cat. Pd, (+ cat. ligand)^a^	temp	**3a**/%^b^	**2a**/%^b^
1	Pd(dba)_2_ (10), XPhos (10)	80	59	22
2	Pd(dba)_2_ (10), PPh_3_ (20)	80	n.d.^c^	<99
3	PEPPSI-IPr (10)	80	7	78
4	XPhos Pd G3 (10)	80	55	7
5	XPhos Pd G4 (10)	80	63	trace
6	XPhos Pd G4 (10)	100	48	14
7	XPhos Pd G4 (10)	40	61	28
8^d^	XPhos Pd G4 (10)	40	48	40
9^e^	XPhos Pd G4 (10)	40	78^f^	4
10^g^	XPhos Pd G4 (10)	40	18	0
11	XPhos Pd G4 (5)	40	58	39
12^e^^,^^h^	XPhos Pd G4 (5)	40	78^f^	n.d.

a Catalyst amount is shown in the parentheses.

b Yields based on ^1^H NMR analysis.

c Not detected.

d The reaction was performed without water.

e The reaction was performed in dioxane and water (v/v = 5/1).

f Isolated yields.

g The reaction was performed in 1,4-dioxane and water (v/v = 1/1).

h The reaction was performed using **1a** (2.0 equiv) at 0.05 M.

A wide range of aryl- and alkenylborons were successfully sulfinylated catalyzed by palladium under the optimized conditions (Figure 2A,B). The reaction using phenylboronic acid pinacol ester took place smoothly to afford sulfoxide **3b** in high yield. Sulfoxides **3c** and **3d** were prepared in moderate yields by 4-methoxyphenylsulfinylation of 2-tolyl- and 2-naphthylboronic acids, respectively. Of note, the sulfinylation of electron-rich 4-methoxy-, 4-hydroxy-, 4-(dimethylamino)-, and 4-(acetylamino)phenylboronic acids proceeded efficiently to provide sulfoxides **3e**–**3h** in good yields, leaving a broad range of electron-donating groups untouched.^9^ Sulfoxides **3i** and **3j** were also synthesized by the sulfinylation of electron-deficient 4-chloro- and 4-acetylphenylboronic acid in moderate to high yields. It is worth noting that we achieved the facile preparation of sulfoxides **3k** and **3l** having a vinyl and a methylthio group, which can be damaged by oxidation in the conventional synthesis. Furthermore, efficient sulfinylation took place to furnish alkenyl sulfoxide **3m** or **3n** when using phenyl- or cyclohexyl-substituted alkenylboronic acid, respectively. This broad substrate scope obviously demonstrated a benefit of the palladium-catalyzed direct sulfinylation of organoborons.

**Figure 2:**
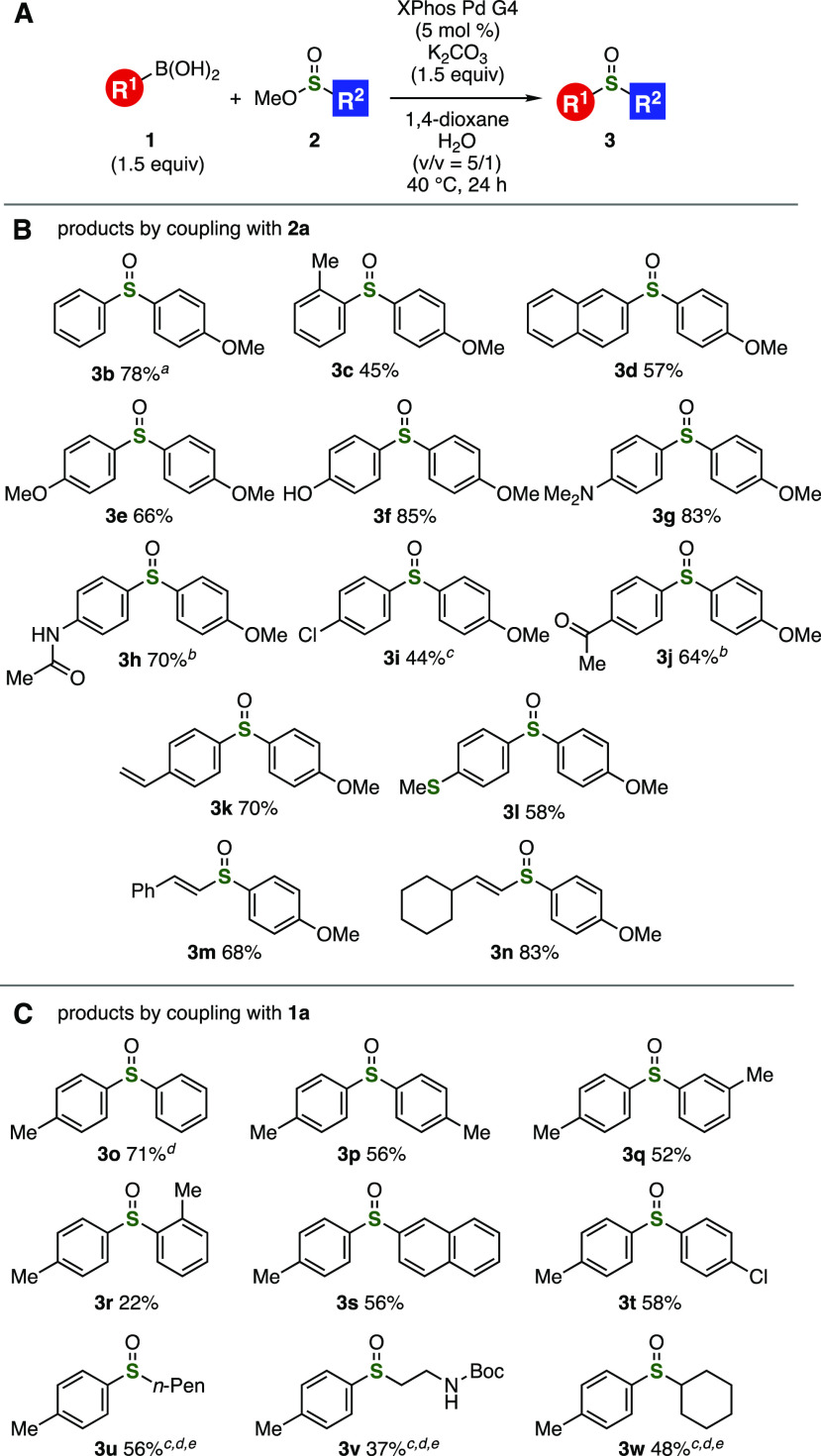
(A) General scheme. (B) Results using various organoborons **1**. (C) Results using various sulfinate esters **2**. See the Supporting Information for the structures of **1** and **2**. ^*a*^Phenylboronic acid pinacol ester was used. ^*b*^XPhos Pd G4 (25 mol %) was used. ^*c*^XPhos Pd G4 (10 mol %) was used. ^*d*^TMEDA (20 mol %) was added. ^*e*^The reaction was performed using **1a** (3.0 equiv) at 60 °C.

Diverse sulfinate esters participated in the catalytic sulfinylation of organoborons allowing us to synthesize a wide variety of sulfoxides **3o**–**3w** (Figures 2A,C).^10^ Phenylation of 4-tolylboronic acid (**1a**) with methyl benzenesulfinate was facilitated by the palladium catalysis to furnish **3o** in good yield, in which the addition of *N,N,N*′,*N*′-tetramethylethylenediamine (TMEDA) slightly improved the efficiency. Sulfoxides **3p** and **3q** were prepared in moderate yields by the reaction using 4- and 3-toluenesulfinic acid methyl esters. Additionally, *S*-tolylation of bulky methyl 2-toluenesulfinate took place albeit in low yield. The palladium-catalyzed sulfinylation with 2-naphthalene- and 4-chlorobenzenesulfinic acid methyl esters was achieved to provide sulfoxides **3s** and **3t** in moderate yields. It is worthy to note that a variety of alkyl aryl sulfoxides **3u**–**3w** were successfully synthesized using primary and secondary sulfinate esters. In particular, we accomplished the catalytic synthesis of sulfoxide **3v** without damaging a (*tert*-butoxycarbonyl)amino group.

To obtain insights into the reaction mechanism, we conducted a number of control experiments (Figure 3). For example, a mixture of **1a** and **2a** was treated with XPhos Pd G4 as a precatalyst in the presence of a catalytic amount of potassium carbonate (Figure 3A). As a result, sulfoxide **3a** was obtained in moderate yield even when using only 5 mol % of base. Treatment of sulfinate ester **2a** with an equimolar amount of XPhos Pd G4 and potassium carbonate followed by the addition of **1a** and potassium carbonate resulted in affording a complex mixture of products, in which sulfoxide **3a** was not detected (Figure 3B, upper). In contrast, sulfoxide **3a** was synthesized in high yield when the palladium precatalyst loading was reduced to 10 mol % (Figure 3B, lower). A plausible reaction mechanism on the basis of these results is illustrated in Figure 3C-a. First, the oxidative addition of sulfinate esters to XPhos-ligated Pd(0) **I** generated in situ would proceed leading to Pd(II) complex **II**.^11,12^ Then, transmetalation between **II** and borates **III** and subsequent reductive elimination will provide sulfoxides, where liberating methoxide from borate **V** can facilitate the reaction. Another mechanism through transmetalation between Pd(II) complex **VI** and borates **III** followed by σ-bond metathesis of Pd(II) intermediate **VII** with sulfinate esters through transition state **VIII** is also possible (Figure 3C-b).^11^ Although further mechanistic studies should be performed to reveal the reaction pathway, it is worth noting that sulfinate esters successfully served as sulfur building blocks without C–S cleavage.^12,13^

**Figure 3:**
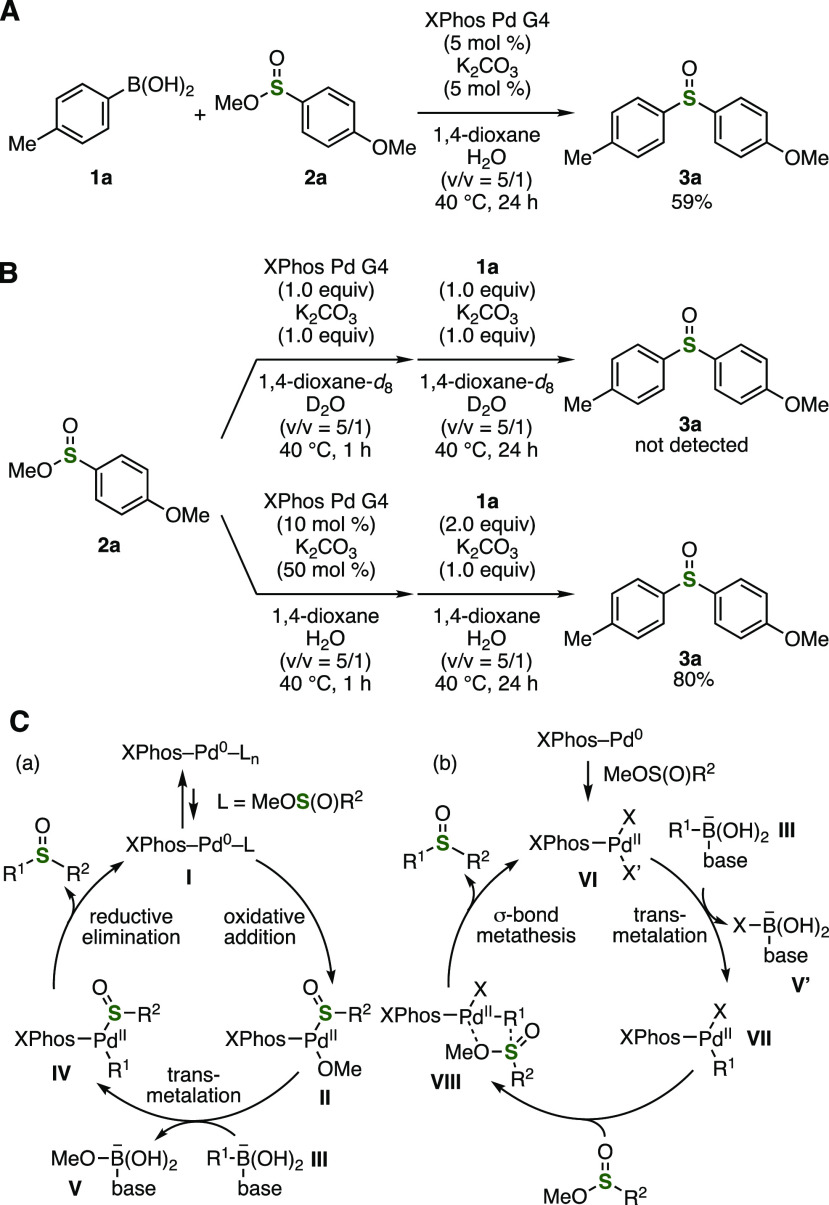
Control experiments and plausible reaction mechanisms. (A) Reaction using a catalytic amount of base. (B) Control experiments from **2a**. (C) Plausible reaction mechanisms. (a) Catalytic cycle via Pd(0) and Pd(II). (b) Catalytic cycle via Pd(II).

An advantage of the palladium-catalyzed sulfoxide synthesis was showcased by consecutive cross-coupling reactions using bromo-substituted sulfinate ester **4** (Figure 4A,B). Bromide-selective Suzuki–Miyaura cross-coupling of **4** catalyzed by palladium with a variety of arylboronic acids proceeded efficiently keeping the sulfinate moiety unreacted (Figure 4A). Then, following *S*-arylation with arylboronic acids realized the synthesis of diverse sulfoxides **6a**–**6d** without damaging hydroxy, formyl, dimethylamino, acetylamino, methylthio, and vinyl groups. Furthermore, we succeeded in the synthesis of sulfoxide **6b** by the consecutive coupling of **4** with arylboronic acids in a one-pot manner (Figure 4B). Since sequential coupling reactions were realized even in the presence of reactive functional groups including formyl and dimethylamino groups owing to the good functional group tolerance, this one-pot procedure will contribute to the modular synthesis of diverse sulfoxides from bromo-substituted sulfinate esters and easily available organoboron derivatives.

**Figure 4:**
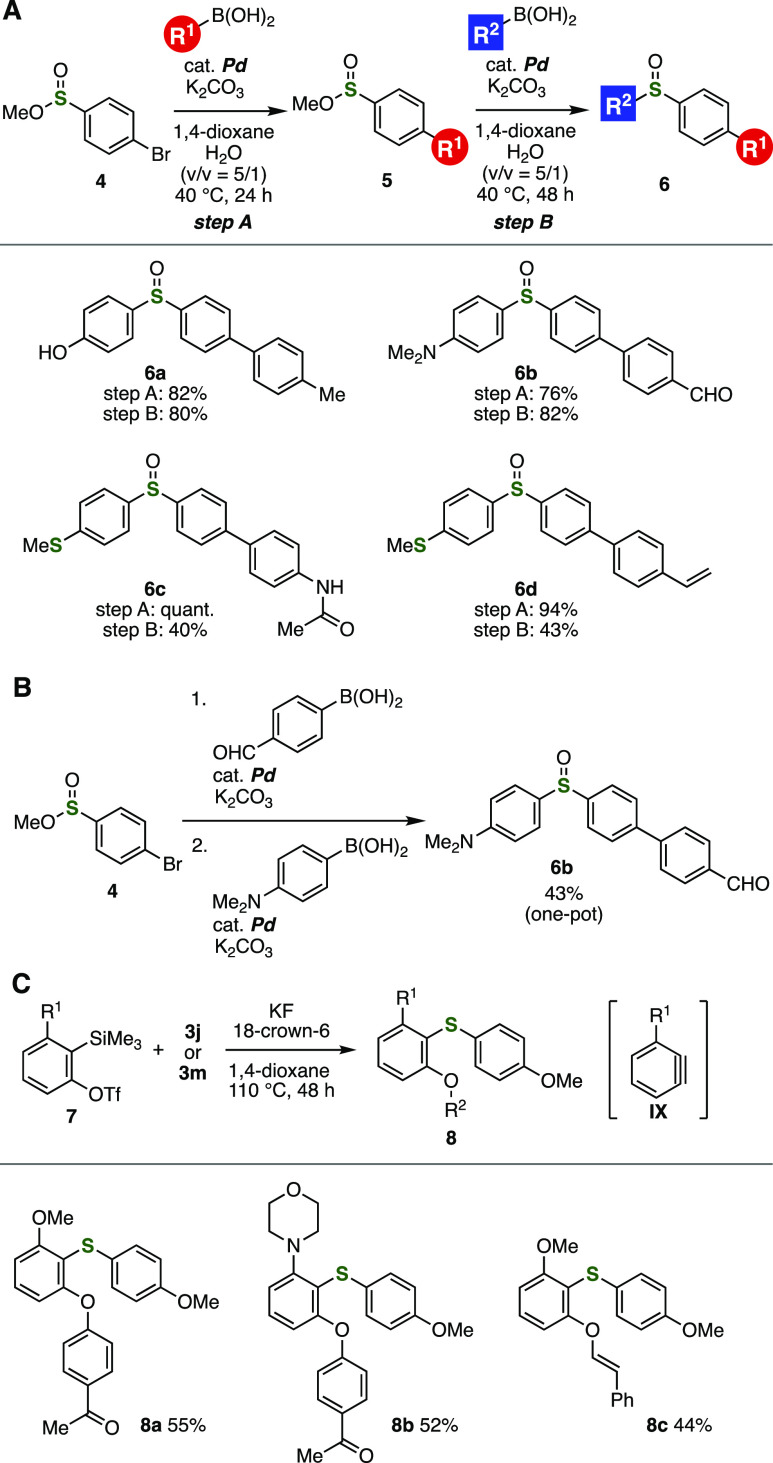
Application of the palladium-catalyzed sulfoxide synthesis. (A) Sequential cross-couplings. (B) One-pot synthesis of sulfoxide **6b**. (C) Aryne reaction of sulfoxide **3j** or **3m**. See the Supporting Information for the details.

The palladium-catalyzed sulfoxide synthesis significantly improved the accessibility of diaryl sulfides by oxythiolation of aryne intermediates **IX** (Figure 4C).^14^ Treatment of *o*-silylaryl triflates **7** and sulfoxide **3j** or **3m** with potassium fluoride and 18-crown-6 in hot dioxane provided a range of diaryl sulfides **8a**–**8c** via selective oxythiolation of arynes **IX** and subsequent *O*-arylation, where an electron-deficient aryl or alkenyl group was selectively migrated. Of note, the synthesis of highly functionalized diaryl sulfides was achieved by virtue of the enhancing the accessibility of sulfoxides developed in this study. Since various functional groups were tolerated in the palladium-catalyzed sulfinylation and this aryne reaction, a modular synthesis of a wide range of diaryl sulfides will be realized from easily available sulfinate esters, organoborons, and *o*-silylaryl triflates.

In summary, we have developed an efficient catalytic method for sulfinylation of organoborons. A wide variety of sulfoxides were synthesized from sulfinate esters and organoborons, keeping easily oxidizable functional groups unreacted. Further studies including detailed mechanistic studies and applications to the synthesis of bioactive organosulfurs are ongoing.

## Supporting Information Available

Experimental procedures and characterization of new compounds including NMR spectra (PDF)
