# Supplementary material for: Palladium-Catalyzed Sulfinylation of Aryl- and Alkenylborons with Sulfinate Esters
Source: Org Lett. 2021 Apr 28;23(9):3793–7. doi: 10.1021/acs.orglett.1c01292 (PMC8289295; doi:10.1021/acs.orglett.1c01292)

Supporting Information

Palladium-Catalyzed Sulfinylation of Aryl- and Alkenylborons with Sulfinate Esters

Minori Suzuki,[†,‡] Kazuya Kanemoto,[†] Yu Nakamura,[†] Takamitsu Hosoya,[†] and Suguru Yoshida*[†,‡]

[†]Laboratory of Chemical Bioscience, Institute of Biomaterials and Bioengineering, Tokyo Medical and Dental University (TMDU), 2-3-10 Kanda-Surugadai, Chiyoda-ku, Tokyo 101-0062, Japan
[‡]Department of Biological Science and Technology, Faculty of Advanced Engineering, Tokyo University of Science, 6-3-1 Niijuku, Katsushika-ku, Tokyo, 125-8585, Japan

Contents

General Information S1
Structures of Organoborons 1 and Sulfinate Esters 2 and 5 S2
Experimental Procedures S3
Characterization Data of New Compounds S7
References for Supporting Information S12
^{1}H and ^{13}C NMR Spectra of Compounds S13

General Information

All reactions were performed with dry glassware under atmosphere of argon, unless otherwise noted. Analytical thin-layer chromatography (TLC) was performed on precoated (0.25 mm) silica-gel plates (Merck Chemicals, Silica Gel 60 F254, Cat. No. 1.05715. Column chromatography was conducted using silica-gel (Kanto Chemical Co., Inc., Silica Gel 60, spherical, particle size 40–50 µm, Cat. No. 37562-85) by conventional manual method. Melting points (Mp) were measured on an OptiMelt MPA100 (Stanford Research Systems), and are uncorrected. ^{1}H NMR spectra were obtained with a Bruker AVANCE 500 spectrometer at 500 MHz. ^{13}C NMR spectra were obtained with a Bruker AVANCE 500 spectrometer at 126 MHz. All NMR measurements were carried out at 25 ºC. $CDCl_3$ (Kanto Chemical Co. Inc., Cat. No. 07663-23) was used as a solvent for obtaining NMR spectra. Chemical shifts (δ) are given in parts per million (ppm) downfield from $(CH_3)_4Si$ (δ 0.00 for ^{1}H NMR in $CDCl_3$) or the solvent peak (δ 77.0 for ^{13}C NMR in $CDCl_3$) as an internal reference with coupling constants (*J*) in hertz (Hz). The abbreviations s, d, t, q, br, and m signify singlet, doublet, triplet, quartet, broad, and multiplet, respectively. IR spectra were measured by diffuse reflectance method on a Shimadzu IRPrestige-21 spectrometer attached with DRS-8000A with the absorption band given in cm^{-1}. High-resolution mass spectra (HRMS) were measured on a Bruker micrOTOF mass spectrometer under positive electrospray ionization (ESI^{+}) conditions.

Unless otherwise noted, materials obtained from commercial suppliers were used without further purification. Methyl *p*-methoxybenzenesulfinate (**2a**),[S1] methyl *p*-toluenesulfinate (**2c**),[S1] methyl *m*-toluenesulfinate (**2d**),[S1] methyl *o*-toluenesulfinate (**2e**),[S1] methyl naphthalene-2-sulfinate (**2f**),[S1] methyl *p*-chlorobenzenesulfinate (**2g**),[S1] methyl pentane-1-sulfinate (**2h**),[S2] methyl 2-(*tert*-butylcarbonylamino)ethyl -1-sulfinate (**2i**),[S3] methyl cyclohexyl sulfinate (**2j**),[S3] and methyl *p*-bromobenzenesulfinate (**4**)[S1] were prepared according to the reported methods.

Structures of Organoborons 1 and Sulfinate Esters 2 and 5

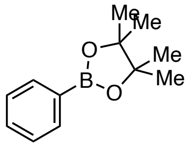

1b'

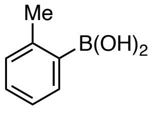

1c

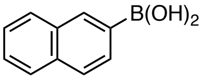

1d

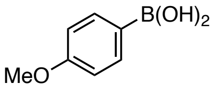

1e

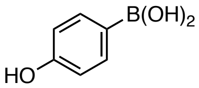

1f

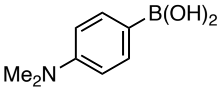

1g

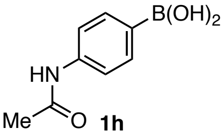

1h

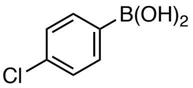

1i

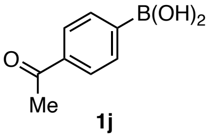

1j

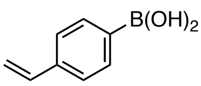

1k

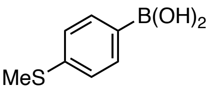

1l

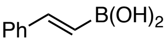

1m

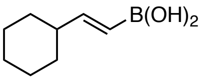

1n

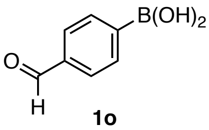

1o

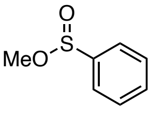

2b

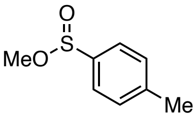

2c

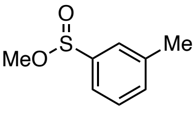

2d

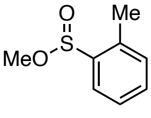

2e

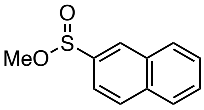

2f

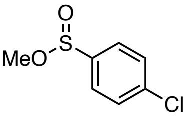

2g

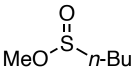

2h

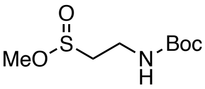

2i

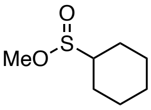

2j

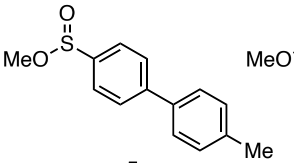

5a

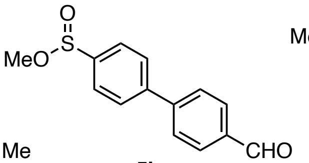

5b

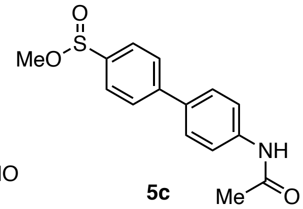

5c

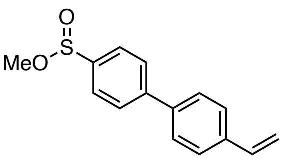

5d

Experimental Procedures

A typical procedure for the palladium-catalyzed sulfinylation of organoborons

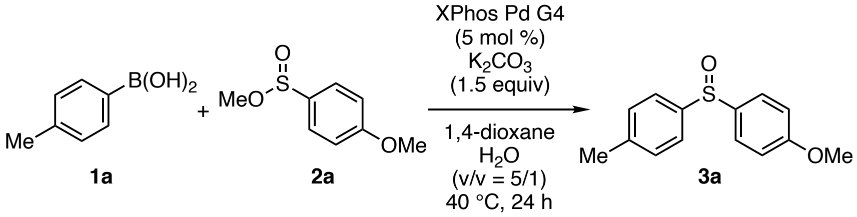

In a 5 mL screw-top V-vial® with a solid-top cap (Sigma-Aldrich, Cat. No. Z115118) were placed *p*-tolylboronic acid (**1a**) (68.5 mg, 0.503 mmol, 2.0 equiv), XPhos Pd G4 (10.8 mg, 12.6 μmol, 5 mol %), and potassium carbonate (51.8 mg, 0.375 mmol, 1.5 equiv). To this was added a solution of methyl *p*-methoxybenzenesulfinate (**2a**) (46.7 mg, 0.251 mmol, 1.0 equiv) dissolved in 1,4-dioxane (4.0 mL) and H_2O (0.8 mL) at room temperature. The mixture was stirred with heating at 40 °C (aluminium heating block) for 24 h. After cooling to room temperature, the mixture was filtered through Na_2SO_4 and washed with EtOAc. The filtrate was concentrated under reduced pressure. The residue was purified by preparative TLC (*n*-hexane/ EtOAc = 1/1) to give *p*-anisyl *p*-tolyl sulfoxide (**3a**) (48.0 mg, 0.148 mmol, 78%) as a yellow oil.

A procedure for the palladium-catalyzed sulfinylation of organoborons at 1mmol scale

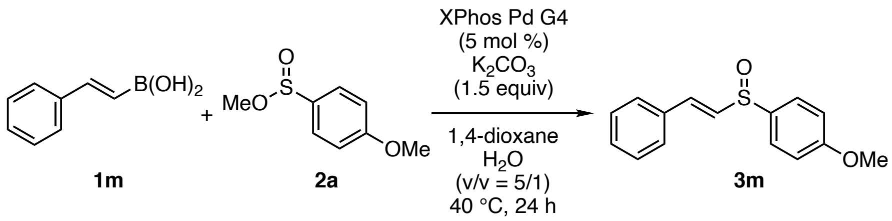

To a mixture of *trans*- β -styrylboronic acid (**1m**) (443.5 mg, 3.00 mmol, 2.0 equiv), XPhos Pd G4 (64.5 mg, 75.0 μmol, 5 mol %), and potassium carbonate (311.4 mg, 2.25 mmol, 1.5 equiv) was added a solution of methyl *p*-methoxybenzenesulfinate (**2a**) (279.1 mg, 1.50 mmol, 1.0 equiv) dissolved in 1,4-dioxane (25.0 mL) and H_2O (5.0 mL) at room temperature. The mixture was stirred with heating at 40 °C (aluminium heating block) for 24 h. After cooling to room temperature, the mixture was added aqueous saturated ammonium chloride. The mixture was extracted with filtered through Na_2SO_4 and washed with EtOAc (30 mL × 3). The combined mixture was washed with brine (10 mL) and dried with Na_2SO_4. After filtration, the filtrate was concentrated under reduced pressure. The residue was purified by flash column chromatography (silica-gel 14 g, *n*-hexane/EtOAc = 10/1 to 3/1) to give *trans*-β-(4-anisylsulfinyl)styrene (**3m**) (336.8 mg, 1.30 mmol, 87%) as a brown oil.

A procedure for the sulfoxide synthesis using a catalytic amount of base

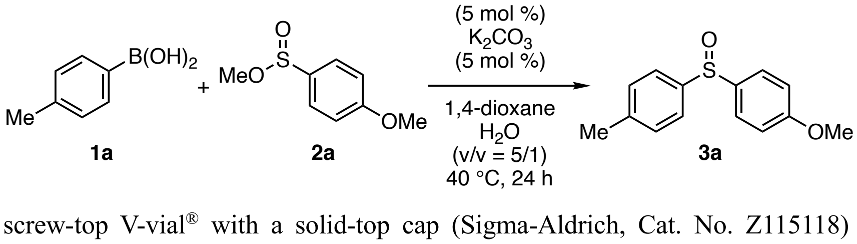

In a 5 mL screw-top V-vial® with a solid-top cap (Sigma-Aldrich, Cat. No. Z115118) were placed *p*-tolylboronic acid (**1a**) (67.8 mg, 0.499 mmol, 2.0 equiv), XPhos Pd G4 (11.1 mg, 12.9 μmol, 5 mol %), and potassium carbonate (1.72 mg, 12.5 μmol, 5 mol %). To this was added a solution of methyl *p*-methoxybenzenesulfinate (**2a**) (46.7 mg, 0.251 mmol, 1.0 equiv) dissolved in 1,4-dioxane (4.0 mL) and H_2O (0.8 mL) at room temperature. The mixture was stirred with heating at 40 °C (aluminium heating block) for 24 h. After cooling to room temperature, the mixture was filtered through Na_2SO_4 and washed with EtOAc. The filtrate was concentrated under reduced pressure. The residue was purified by preparative TLC (*n*-hexane/ EtOAc = 1/1) to give *p*-anisyl *p*-tolyl sulfoxide (**3a**) (36.5 mg, 0.148 mmol, 59%) as a yellow oil.

A control experiment for the sulfoxide synthesis using an equimolar amount of XPhos Pd G4

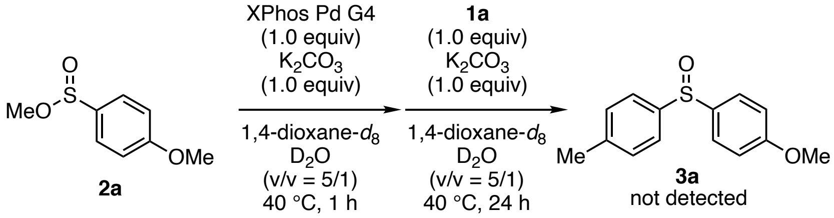

In a 5 mL screw-top V-vial® with an open-top cap (Sigma-Aldrich, Cat. No. Z115150) were placed methyl *p*-methoxybenzenesulfinate (**2a**) (9.5 mg, 51 μmol, 1.0 equiv), XPhos Pd G4 (42.8 mg, 49.8 μmol, 1.0 equiv), 1,4-dioxane-d_8 (0.83 mL), and D_2O (0.17 mL) at room temperature. Then, potassium carbonate (7.2 mg, 52 μmol, 1.0 equiv) was added to the mixture at room temperature. After stirring at 40 °C for 1 h, *p*-tolylboronic acid (**1a**) (6.9 mg, 51 μmol, 1.0 equiv) and potassium carbonate (7.1 mg, 51 μmol, 1.0 equiv). After stirring at 40 °C for 1 h, the reaction was monitored by ^{1}H NMR analysis using a small amount of the aliquot from the mixture, in which sulfoxide **3a** was not detected. After further stirring at 40 °C for 23 h, the mixture was filtered through Na_2SO_4 and washed with EtOAc. The filtrate was concentrated under reduced pressure. When the residue was purified by preparative TLC (*n*-hexane/ EtOAc = 1/1), sulfoxide **3a** was not obtained.

A control experiment for the sulfoxide synthesis using a catalytic amount of XPhos Pd G4

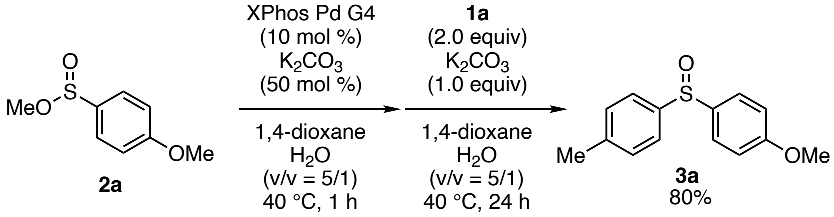

In a 5 mL screw-top V-vial® with a solid-top cap (Sigma-Aldrich, Cat. No. Z115118) were placed methyl *p*-methoxybenzenesulfinate (**2a**) (47.9 mg, 0.256 mmol, 1.0 equiv), XPhos Pd G4 (42.8 mg, 25.1 μmol, 10 mol %), 1,4-dioxane (4.0 mL), and H_2O (0.8 mL) at room temperature. Then, potassium carbonate (17.5 mg, 0.127 mmol, 50 mol %) was added to the mixture at room temperature. After stirring at 40 °C for 1 h, *p*-tolylboronic acid (**1a**) (68.3 mg, 0.502 mmol, 2.0 equiv) and potassium carbonate (34.9 mg, 0.253 mmol, 1.0 equiv). After further stirring at 40 °C for 24 h, the mixture was filtered through Na_2SO_4 and washed with EtOAc. The filtrate was concentrated under reduced pressure. The residue was purified by preparative TLC (*n*-hexane/ EtOAc = 1/1) to give *p*-anisyl *p*-tolyl sulfoxide (**3a**) (50.4 mg, 0.205 mmol, 80%) as a yellow oil.

Detailed conditions for the sequential cross-coupling reactions

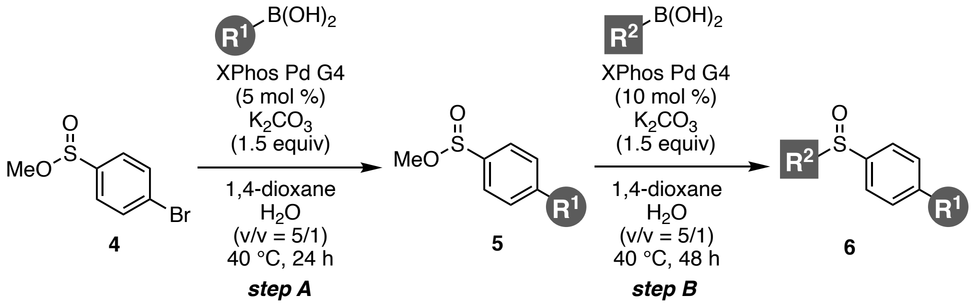

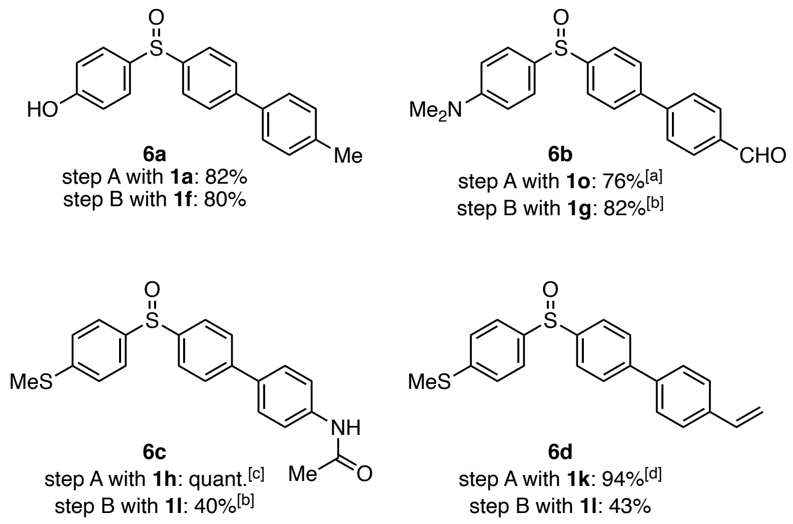

[a] The reaction was performed with XPhos Pd G4 (10 mol %) for 3.5 h.
[b] The reaction was performed with XPhos Pd G4 (5 mol %).
[c] The reaction was performed with XPhos Pd G4 (10 mol %) for 7 h.
[d] The reaction was performed with XPhos Pd G4 (10 mol %) for 24 h.

*A typical procedure for the Suzuki–Miyaura coupling of methyl 4-bromobenzenesulfinate (**4**)*

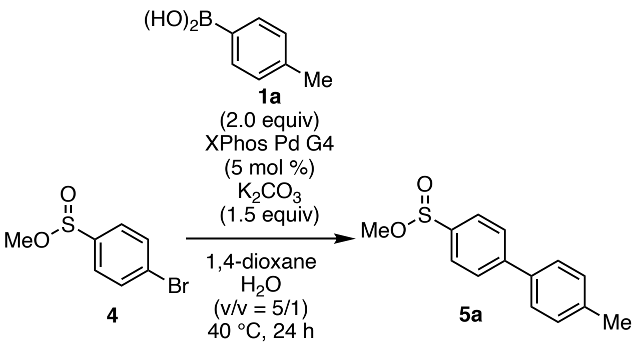

To a mixture of *p*-tolylboronic acid (**1a**) (408 mg, 3.00 mmol, 2.0 equiv), XPhos Pd G4 (64.2 mg, 0.747 mmol, 5 mol %), and potassium carbonate (311 mg, 2.25 mmol, 1.5 equiv) was added a solution of methyl *p*-bromobenzenesulfinate (**4**) (352 mg, 1.50 mmol, 1.0 equiv) dissolved in 1,4-dioxane (2.5 mL) and H_2O (0.5 mL) at room temperature. The mixture was stirred with heating at 40 °C (oil bath) for 24 h. After cooling to room temperature, to the mixture was added aqueous saturated ammonium chloride (20 mL). The mixture was extracted with EtOAc (20 mL × 3). The combined mixture was washed with brine (10 mL) and dried with Na_2SO_4. After filtration, the filtrate was concentrated under reduced pressure. The residue was purified by flash column chromatography (silica-gel 12 g, *n*-hexane/EtOAc = 5/1) to give methyl *p*-(*p*-tolyl)benzenesulfinate (**5a**) (303 mg, 1.23 mmol, 82%) as an orange solid.

*A typical procedure for the consecutive cross-couplings of methyl 4-bromobenzenesulfinate (**4**)*

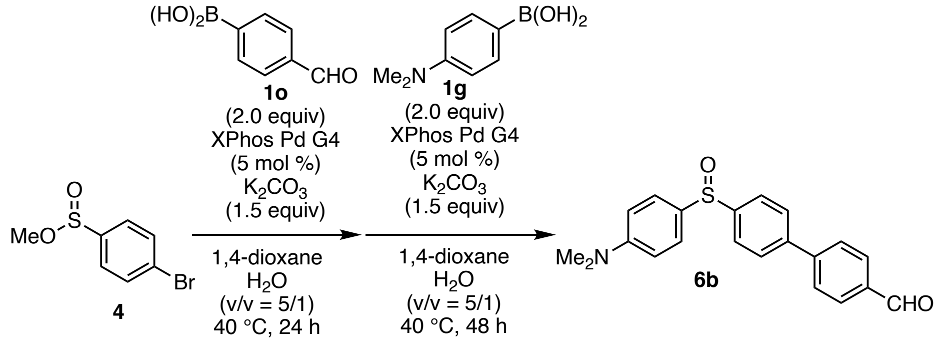

In a 5 mL screw-top V-vial® with a solid-top cap (Sigma-Aldrich, Cat. No. Z115118) were placed *p*-formylphenylboronic acid (**1o**) (45.2 mg, 0.302 mmol, 2.0 equiv), XPhos Pd G4 (6.5 mg, 7.6 µmol, 5 mol %), and potassium carbonate (31.4 mg, 0.228 mmol, 1.5 equiv). To this was added a solution of methyl *p*-bromobenzenesulfinate (**4**) (35.9 mg, 0.153 mmol, 1.0 equiv) dissolved in 1,4-dioxane (2.5 mL) and H_2O (0.5 mL) at room temperature. The mixture was stirred with heating at 40 °C (aluminium heating block) for 24 h. Then, to the resulting mixture were added *p*-(dimethylamino)phenylboronic acid (**1g**) (49.2 mg, 0.298 mmol, 2.0 equiv), XPhos Pd G4 (6.4 mg, 7.4 µmol, 5 mol %), and potassium carbonate (31.4 mg, 0.228 mmol, 1.5 equiv). The mixture was stirred with heating at 40 °C (aluminium heating block) for 48 h. After cooling to room temperature, the mixture was filtered through Na_2SO_4 and washed with EtOAc. The filtrate was concentrated under reduced pressure. The residue was purified by preparative TLC (dichloromethane/methanol = 15/1) to give *p*-(dimethylamino)phenyl *p*-tolylphenyl sulfoxide (**6b**) (23.0 mg, 65.8 µmol, 43%) as a yellow sold.

*A typical procedure for preparation of o-arylthio-substituted diaryl ethers **8***

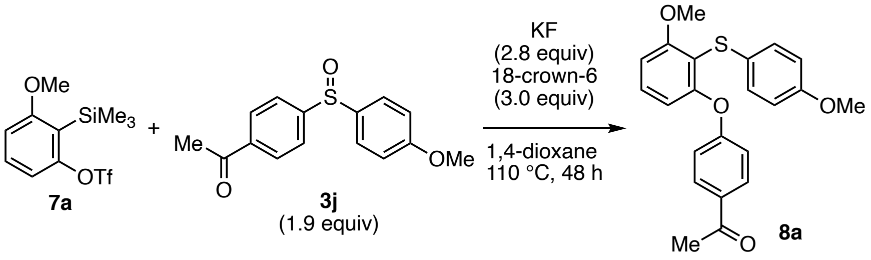

In a 5 mL screw-top V-vial® with a solid-top cap (Sigma-Aldrich, Cat. No. Z115118) were placed *o*-(trimethylsilyl)phenyl triflate (**7a**) (17.0 mg, 51.8 µmol, 1.0 equiv) and *p*-acetylphenyl *p*-anisyl sulfoxide (**3j**) (26.9 mg, 98.2 µmol, 1.9 equiv) dissolved in 1,4-dioxane (1.0 mL). To this were added 18-crown-6 (40.8 mg, 0.155 mmol, 3.0 equiv) and potassium carbonate (8.3 mg, 0.14 mmol, 2.8 equiv) at room temperature. The mixture was stirred with heating at 110 °C (aluminium heating block) for 48 h. Then, to the resulting mixture was added H_2O (3 mL). The mixture was extracted with EtOAc (5 mL × 3). The combined mixture was dried with Na_2SO_4. After filtration, the filtrate was concentrated under reduced pressure. The residue was purified by preparative TLC (*n*-hexane/EtOAc = 3/1) to give *p*-acetylphenyl 3-methoxy-2-(phenylthiol)phenyl ether (**8a**) (10.8 mg, 28.5 µmol, 55%) as colorless oil.

Similarly, dialyl ethers **8b** and **8c** were prepared from the corresponding *o*-silylaryl triflates and sulfoxides.

Characterization Data of New Compounds

4-Anisyl 4-tolyl sulfoxide (**3a**),[S4] 4-anisyl phenyl sulfoxide (**3b**),[S4] 4-anisyl 2-tolyl sulfoxide (**3c**),[S4] 4-anisyl 2-naphthyl sulfoxide (**3d**),[S5] bis(4-anisyl) sulfoxide (**3e**),[S4] 4-anisyl 4-hydroxyphenyl sulfoxide (**3f**),[S4] 4-anisyl 4-(dimethylamino)phenyl sulfoxide (**3i**),[S4] *trans*- *β* -(4-anisylsulfinyl)styrene (**3m**),[S6] phenyl 4-tolyl sulfoxide (**3o**),[S4] bis(4-tolyl) sulfoxide (**3p**),[S4] 3-tolyl 4-tolyl sulfoxide (**3q**),[S4] 2-tolyl 4-tolyl sulfoxide (**3r**),[S7] 4-tolyl 2-naphthyl sulfoxide (**3s**),[S5] 4-chlorophenyl 4-tolyl sulfoxide (**3t**),[S4] *n*-pentyl 4-tolyl sulfoxide (**3u**),[S8] and 2-(*tert*-butoxycarbonylamino)ethyl 4-tolyl sulfoxide (**3w**)[S9] were identical in spectra data with those reported in the literature.

4-Anisyl 4-(dimethylamino)phenyl sulfoxide (**3g**)

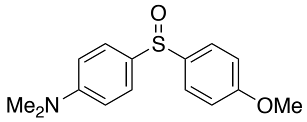

Yield: 83% (57.8 mg, 0.210 mmol); Colorless solid; Mp 101–103 °C; TLC R_f 0.43 (*n*-hexane/EtOAc = 2/3); ^{1}H NMR ($CDCl_3$, 400 MHz): δ 7.55–7. 49 (AA'BB', 2H), 7.47–7.41 (AA'BB', 2H), 6.97–6.92 (AA'BB', 2H), 6.71–6.66 (AA'BB', 2H), 3.82 (s, 3H), 2.99 (s, 6H); ^{13}C NMR ($CDCl_3$, 126 MHz): δ 161.6 (1C), 152.5 (1C), 137.7 (1C), 131.3 (1C), 127.6 (2C), 126.9 (2C), 114.7 (2C), 112.1 (2C), 55.7 (1C), 40.4 (2C); IR (KBr, cm^{-1}) 1030, 1080, 1091, 1250, 1364, 1495, 1508, 1593; HRMS (ESI) *m*/*z*: $[M+H]^+$ Calcd for $C_{15}H_{18}NO_2S^+$ 276.1053; Found 276.1052.

4-(Acetylamino)phenyl 4-anisyl sulfoxide (**3h**)

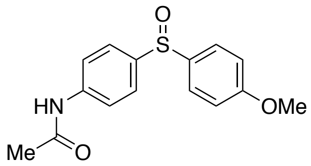

Yield: 70% (50.5 mg, 0.175 mmol); Yellow oil; TLC R_f 0.38 (CH_2Cl_2/MeOH = 15/1); ^{1}H NMR ($CDCl_3$, 500 MHz): δ 7.74–7.46 (m, 6H), 7.04–6.90 (AA'BB', 2H), 3.82 (s, 3H), 2.15 (s, 3H); ^{13}C NMR ($CDCl_3$, 126 MHz): δ 169.2 (1C), 162.3 (1C), 141.2 (1C), 139.9 (1C), 136.4 (1C), 127.3 (2C), 126.1 (2C), 120.4 (2C), 115.1 (2C), 55.7 (1C), 24.8 (1C); IR (KBr, cm^{-1}) 767, 797, 827, 1026, 1080, 1089, 1258, 1312, 1398, 1495, 1533, 1692; HRMS (ESI) *m*/*z*: $[M+Na]^+$ Calcd for $C_{15}H_{15}NO_3SNa^+$ 312.0665; Found 312.0673.

4-Acetylphenyl 4-anisyl sulfoxide (**3j**)

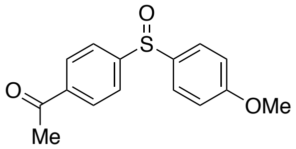

Yield: 64% (44.6 mg, 0.163 mmol); Orange solid; Mp 154–156 °C; TLC R_f 0.30 (*n*-hexane/EtOAc = 1/1); ^{1}H NMR ($CDCl_3$, 500 MHz): δ 8.11–8.00 (AA'BB', 2H), 7.78–7.68 (AA'BB', 2H), 7.67–7.56 (AA'BB', 2H), 7.06–6.94 (AA'BB', 2H), 3.85 (s, 3H), 2.61 (s, 3H); ^{13}C NMR ($CDCl_3$, 126 MHz): δ 197.3(1C), 162.6 (1C), 151.2 (1C), 138.9 (1C), 136.3 (1C), 129.2 (2C), 127.7 (2C), 124.8 (2C), 115.3 (2C), 55.8 (1C), 27.0 (1C); IR (KBr, cm^{-1}) 669, 772, 827, 1032, 1043, 1219, 1258, 1495, 1593, 1686, 3019; HRMS (ESI) *m*/*z*: $[M+Na]^+$ Calcd for $C_{15}H_{14}O_3SNa^+$ 297.0556; Found 297.0562.

4-Anisyl 4-vinylphenyl sulfoxide (**3k**)

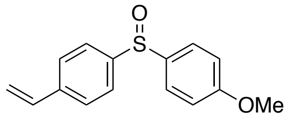

Yield: 70% (44.9 mg, 0.174 mmol); Colorless oil; TLC R_f 0.60 (*n*-hexane/EtOAc = 1/1); ^{1}H NMR ($CDCl_3$, 400 MHz): δ 7.58–7.54 (m, 4H), 7.49–7.46 (AA'BB', 2H), 6.98–6.93 (AA'BB', 2H), 6.74–6.67 (dd, 1H, J = 10.9, 17.6 Hz), 5.82–5.77 (d, 1H, J = 17.6 Hz), 5.35–5.32 (d, 1H, J = 10.9 Hz), 3.81 (s, 3H); ^{13}C NMR ($CDCl_3$, 126 MHz): δ 162.3 (1C), 145.1 (1C), 140.3 (1C), 137.0 (1C), 135.9 (1C), 127.4 (2C), 127.2 (2C), 125.1 (2C), 116.3 (1C), 115.1 (2C), 55.7 (1C); IR (KBr, cm^{-1}) 536, 829, 1028, 1043, 1090, 1252, 1493, 1591; HRMS (ESI) *m*/*z*: $[M+H]^+$ Calcd for $C_{15}H_{15}O_2S^+$ 259.0788; Found 259.0794.

4-Anisyl 4-(methylthio)phenyl sulfoxide (**3l**)

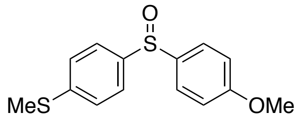

Yield: 58% (40.1 mg, 0.144 mmol); Colorless solid; Mp 87–90 °C; TLC R_f 0.40 (*n*-hexane/EtOAc = 1/1); ^{1}H NMR ($CDCl_3$, 400 MHz): δ 7.57–7.53 (AA'BB', 2H), 7.52–7.48 (AA'BB', 2H), 7.30–7.26 (AA'BB', 2H), 6.97–6.95 (AA'BB', 2H), 3.82 (s, 3H), 2.48 (s, 3H); ^{13}C NMR ($CDCl_3$, 126 MHz): δ 162.2 (1C), 143.1 (1C), 142.1 (1C), 137.0 (1C), 127.3 (2C), 126.4 (2C), 125.4 (2C), 115.1 (2C), 55.7 (1C), 15.4 (1C); IR (KBr, cm^{-1}) 552, 743, 912, 1033, 1083, 1254, 1476, 1593, 2941; HRMS (ESI) *m*/*z*: [M+H]$^+$ Calcd for $C_{14}H_{15}O_2S_2^+$ 279.0508; Found 279.0501.

trans-2-Cyclohexylvinyl 4-methoxyphenyl sulfoxide (**3n**)

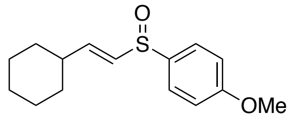

Yield: 83% (62.9 mg, 0.238 mmol); Colorless oil; TLC R_f 0.47 (*n*-hexane/EtOAc = 1/1); ^{1}H NMR ($CDCl_3$, 500 MHz): δ 7.58–7.53 (AA'BB', 2H), 7.04–6.99 (AA'BB', 2H), 6.56 (dd, 1H, J = 6.6, 15.3 Hz), 6.15 (dd, 1H, J = 1.3, 15.3 Hz), 3.86 (s, 3H), 2.22–2.14 (m, 1H), 1.83–1.65 (m, 5H), 1.34–1.15 (m, 5H); ^{13}C NMR ($CDCl_3$, 126 MHz): δ 162.0 (1C), 145.6 (1C), 135.6 (1C), 133.1 (1C), 126.9 (2C), 115.0 (2C), 55.7 (1C), 40.5 (1C), 32.0 (2C), 26.0 (1C), 25.9 (2C); IR (KBr, cm^{-1}) 772, 829, 1030, 1086, 1217, 1252, 1304, 1495, 1593, 2853, 2926; HRMS (ESI) *m*/*z*: [M+H]$^+$ Calcd for $C_{15}H_{21}O_2S^+$ 265.1257; Found 265.1257.

2-(*tert*-Butoxycarbonylamino)ethyl 4-tolyl sulfoxide (**3v**)

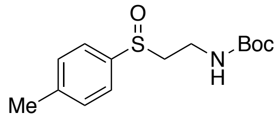

Yield: 37% (26.4 mg, 98.7 μmol); Colorless oil; TLC R_f 0.26 (*n*-hexane/EtOAc = 2/3); ^{1}H NMR ($CDCl_3$, 500 MHz): δ 7.52–7.49 (AA'BB', 2H), 7.36–7.32 (AA'BB', 2H), 5.20 (br s, 1H), 3.63–3.48 (m, 2H), 3.16–3.08 (m, 1H), 2.87–2.81 (m, 1H), 2.42 (s, 3H), 1.43 (s, 9H); ^{13}C NMR ($CDCl_3$, 126 MHz): δ 156.02 (1C), 156.00 (1C), 141.9 (1C), 130.4 (2C), 124.2 (2C), 79.9 (1C), 35.21 (1C), 35.19 (1C), 28.6 (3C), 21.6 (1C); IR (KBr, cm^{-1}) 770, 1015, 1026, 1040, 1171, 1252, 1271, 1366, 1495, 1512, 1707; HRMS (ESI) *m*/*z*: [M+Na]$^+$ Calcd for $C_{14}H_{21}NO_3SNa^+$ 306.1134; Found 306.1134.

Methyl 4-(4-tolyl)benzenesulfinate (**5a**)

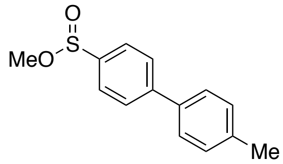

Yield: 82% (321 mg, 1.23 mmol); Orange solid; Mp 73–75 °C; TLC R_f 0.50 (*n*-hexane/EtOAc = 5/1); ^{1}H NMR ($CDCl_3$, 500 MHz): δ 7.80–7.75 (m, 4H), 7.56–7.52 (AA'BB', 2H), 7.33–7.30 (AA'BB', 2H), 3.55 (s, 3H), 2.44 (s, 3H); ^{13}C NMR ($CDCl_3$, 126 MHz): δ 145.4 (1C), 142.5 (1C), 138.5 (1C), 137.0 (1C), 130.0 (2C), 127.8 (2C), 127.4 (2C), 126.1 (2C), 49.9 (1C), 21.4 (1C); IR (KBr, cm^{-1}) 669, 772, 808, 964, 1126, 1219, 3019; HRMS (ESI) *m*/*z*: [M+Na]$^+$ Calcd for $C_{14}H_{14}O_2SNa^+$ 269.0607; Found 269.0599.

Methyl 4-(4-formylphenyl)benzenesulfinate (**5b**)

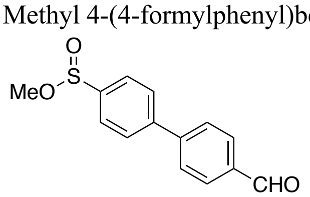

Yield: 76% (299 mg, 1.15 mmol); Yellow solid; Mp 77–79 °C; TLC R_f 0.31 (*n*-hexane/EtOAc = 3/1); ^{1}H NMR ($CDCl_3$, 400 MHz): δ 10.1 (s, 1H), 8.02–7.98 (AA'BB', 2H), 7.85–7.77 (m, 6H), 3.55 (s, 3H); ^{13}C NMR ($CDCl_3$, 126 MHz): δ 191.9 (1C), 145.7 (1C), 144.1 (1C), 143.9 (1C), 136.1 (1C), 130.6 (2C), 128.3 (2C), 128.2 (2C), 126.4 (2C), 50.1 (1C); IR (KBr, cm^{-1}) 667, 687, 772, 818, 964, 1128, 1171, 1217, 1607, 1703, 2399, 3019; HRMS (ESI) *m*/*z*: [M+Na]$^+$ Calcd for $C_{14}H_{12}O_3SNa^+$ 283.0399; Found 283.0398.

Methyl 4-(4-(acetylamino)phenyl)benzenesulfinate (**5c**)

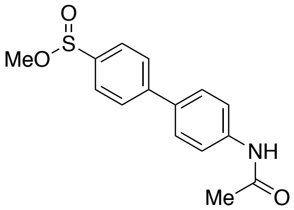

Yield: quant. (345mg, 1.19 mmol); Colorless solid; Mp 157–159 °C; TLC R_f 0.49 (CH_2Cl_2/MeOH = 15/1); ^{1}H NMR ($CDCl_3$, 500 MHz): δ 7.80–7.71 (m, 4H), 7.66–7.56 (m, 4H), 3.53 (s, 3H), 2.22 (s, 3H); ^{13}C NMR ($CDCl_3$, 126 MHz): δ 168.5 (1C), 144.7 (1C), 142.6 (1C), 138.3 (1C), 135.7 (1C), 128.1 (2C), 127.6 (2C), 126.2 (2C), 120.4 (2C), 49.9 (1C), 24.9 (1C); IR (KBr, cm^{-1}) 673, 718, 772, 818, 1219, 1533, 1667; HRMS (ESI) *m*/*z*: $[M+Na]^+$ Calcd for $C_{15}H_{15}NO_3SNa^+$ 312.0665; Found 312.0667.

Methyl 4-(4-vinylphenyl)benzenesulfinate (**5d**)

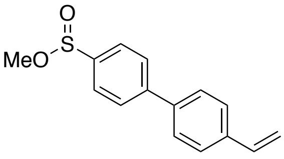

Yield: 94% (292 mg, 1.13 mmol); Orange solid; Mp 76–79 °C; TLC R_f 0.46 (*n*-hexane/EtOAc = 5/1); ^{1}H NMR ($CDCl_3$, 500 MHz): δ 7.82–7.68 (m, 4H), 7.62–7.60 (AA'BB', 2H), 7.48–7.46 (AA'BB', 2H), 6.79 (dd, 1H, *J* = 10.9, 17.6 Hz), 5.85 (d, 1H, *J* = 17.6 Hz), 5.35 (d, 1H, *J* = 10.9 Hz), 3.56 (s, 3H); ^{13}C NMR ($CDCl_3$, 126 MHz): δ 144.9 (1C), 142.8 (1C), 139.1 (1C), 137.8 (1C), 136.3 (1C), 127.8 (2C), 127.7 (2C), 127.1 (2C), 126.2 (2C), 114.9 (1C), 49.9 (1C); IR (KBr, cm^{-1}) 627, 669, 685, 772, 824, 928, 1219, 1520, 3019; HRMS (ESI) *m*/*z*: $[M+Na]^+$ Calcd for $C_{15}H_{14}O_2SNa^+$ 281.0607; Found 281.0611.

4-Hydroxyphenyl 4-(4-tolyl)phenyl sulfoxide (**6a**)

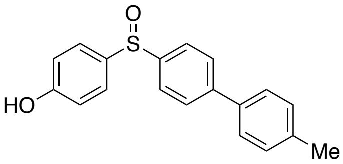

Yield: 80% (24.9 mg, 80.7 μmol); Brown solid; Mp 196–198 °C; TLC R_f 0.32 (*n*-hexane/EtOAc = 1/1); ^{1}H NMR ($CDCl_3$, 500 MHz): δ 7.68–7.62 (m, 4H), 7.52–7.48 (AA'BB', 2H), 7.48–7.45 (AA'BB', 2H), 7.27–7.24 (AA'BB', 2H), 6.91–6.87 (AA'BB', 2H), 2.40 (s, 3H); ^{13}C NMR ($CDCl_3$, 126 MHz): δ 159.5 (1C), 144.1 (1C), 143.4 (1C), 138.3 (1C), 137.0 (1C), 135.8 (1C), 129.9 (2C), 128.2 (2C), 128.0 (2C), 127.3 (2C), 125.5 (2C), 116.9 (2C), 21.4 (1C); IR (KBr, cm^{-1}) 758, 806, 833, 1001, 1020, 1088, 1261, 1281, 1584, 3021, 3061, 3107; HRMS (ESI) *m*/*z*: $[M+Na]^+$ Calcd for $C_{19}H_{16}O_2SNa^+$ 331.0763; Found 331.0753.

4-(Dimethylamino)phenyl 4-(4-formylphenyl)phenyl sulfoxide (**6b**)

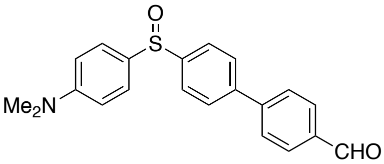

Yield: 82% (28.5 mg, 81.6 μmol); Yellow solid; Mp 143–145 °C; TLC R_f 0.56 (CH_2Cl_2/MeOH = 15/1); ^{1}H NMR ($CDCl_3$, 500 MHz): δ 10.1 (s, 1H), 8.00–7.96 (AA'BB', 2H), 7.78–7.74 (AA'BB', 2H), 7.74–7.70 (m, 4H), 7.55–7.51 (AA'BB', 2H), 6.75–6.70 (AA'BB', 2H), 3.02 (s, 6H); ^{13}C NMR ($CDCl_3$, 126 MHz): δ 192.0 (1C), 152.7 (1C), 146.7 (1C), 146.1 (1C), 146.1 (1C), 141.8 (1C), 135.8 (1C), 130.6 (2C), 130.5 (2C), 128.1 (2C), 128.0 (2C), 125.4 (2C), 112.2 (2C), 40.3 (2C); IR (KBr, cm^{-1}) 772, 814, 1003, 1037, 1092, 1219, 1366, 1512, 1593, 1699; HRMS (ESI) *m*/*z*: $[M+H]^+$ Calcd for $C_{21}H_{20}NO_2S^+$ 350.1209; Found 350.1209.

4-(4-(Acetylamino)phenyl) 4-(methylthio)phenyl sulfoxide (**6c**)

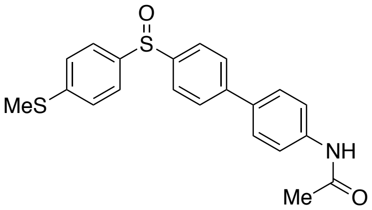

Yield: 40% (15.3 mg, 40.1 μmol); Colorless solid; Mp 194–196 °C; TLC *R*f 0.44 (CH_2Cl_2/MeOH = 15/1); ^{1}H NMR ($CDCl_3$, 400 MHz): δ 7.69–7.61 (m, 4H), 7.61–7.50 (m, 6H), 7.32–7.28 (AA'BB', 2H), 2.49 (s, 3H), 2.21 (s, 3H); ^{13}C NMR ($CDCl_3$, 126 MHz): δ 168.7 (1C), 144.1 (1C), 143.7 (1C), 143.6 (1C), 141.6 (1C), 138.3 (1C), 135.6 (1C), 127.9 (2C), 127.9 (2C), 126.5 (2C), 125.6 (2C), 125.6 (2C), 120.4 (2C), 24.9 (1C), 15.3 (1C); IR (KBr, cm^{-1}) 627, 669, 772, 928, 1032, 1043, 1217, 1423, 1522, 1686, 2976, 3019; HRMS (ESI) *m*/*z*: [M+Na]$^+$ Calcd for $C_{21}H_{19}NO_2S_2Na^+$ 404.0749; Found 404.0759.

4-(Methylthio)phenyl 4-(4-vinylphenyl)phenyl sulfoxide (**6d**)

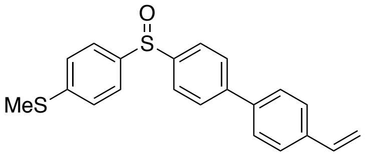

Yield: 43% (14.8 mg, 42.2 μmol); Colorless solid; Mp 133–136 °C; TLC *R*f 0.35 (*n*-hexane/EtOAc = 2/1); ^{1}H NMR ($CDCl_3$, 400 MHz): δ 7.70–7.65 (m, 4H), 7.60–7.55 (AA'BB', 2H), 7.55–7.51 (AA'BB', 2H), 7.50–7.47 (AA'BB', 2H), 7.32–7.28 (AA'BB', 2H), 6.75 (dd, 1H, *J* = 10.9, 17.6 Hz), 5.80 (d, 1H, *J* = 17.6 Hz), 5.30 (d, 1H, *J* = 10.9 Hz), 2.49 (s, 3H); ^{13}C NMR ($CDCl_3$, 126 MHz): δ 144.5 (1C), 143.8 (1C), 143.6 (1C), 141.7 (1C), 139.2 (1C), 137.7 (1C), 136.4 (1C), 128.0 (2C), 127.5 (2C), 127.0 (2C), 126.5 (2C), 125.6 (2C), 125.5 (2C), 114.8 (1C), 15.3 (1C); IR (KBr, cm^{-1}) 557, 743, 754, 810, 824, 1043, 1070, 1084, 1391, 1476, 1574; HRMS (ESI) *m*/*z*: [M+Na]$^+$ Calcd for $C_{21}H_{18}OS_2Na^+$ 373.0691; Found 373.0690.

4-Acetylphenyl 2-(4-anisylthio)-3-methoxyphenyl ether (**8a**)

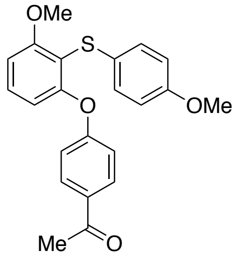

Yield: 55% (10.8 mg, 28.3 μmol); Colorless oil; TLC *R*f 0.35 (*n*-hexane/EtOAc = 3/1); ^{1}H NMR ($CDCl_3$, 400 MHz): δ 7.88–7.83 (AA'BB', 2H), 7.34 (dd, 1H, *J* = 8.4, 8.4 Hz), 7.17–7.12 (AA'BB', 2H), 6.78–6.84 (m, 3H), 6.72–6.67 (m, 3H), 3.88 (s, 3H), 3.73 (s, 3H), 2.55 (s, 3H); ^{13}C NMR ($CDCl_3$, 126 MHz): δ 196.9, 162.1, 161.5, 158.7, 157.0, 131.9, 131.7, 130.7, 130.6, 127.1, 116.8, 114.4, 114.3, 108.3, 56.6, 55.5, 26.6 (One overlapped peak was not determined.); IR (KBr, cm^{-1}) 1076, 1165, 1242, 1269, 1435, 1364, 1493, 1572, 1584, 1599, 1678; HRMS (ESI) *m*/*z*: [M+Na]$^+$ Calcd for $C_{22}H_{20}O_4SNa^+$ 403.0975; Found 403.0975.

The regiochemistry was determined by the HMBC experiment.

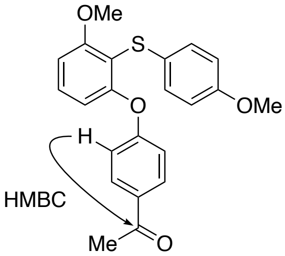

4-Acetylphenyl 2-(4-anisylthio)-3-morpholinophenyl ether (**8b**)

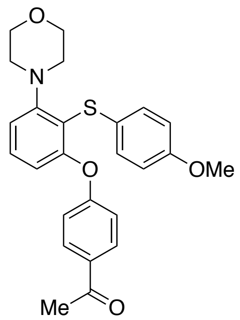

Yield: 52% (11.7 mg, 26.9 μmol); Colorless oil; TLC R_f 0.40 (*n*-hexane/EtOAc = 2/1); ^{1}H NMR ($CDCl_3$, 500 MHz): δ 7.88–7.82 (AA'BB', 2H), 7.32 (dd, 1H, J = 8.0, 8.0 Hz), 7.09–7.03 (AA'BB', 2H), 6.93 (d, 1H, J = 8.0 Hz), 6.80–6.68 (m, 5H), 3.75–3.68 (m, 7H), 3.11–3.07 (m, 4H), 2.54 (s, 3H); ^{13}C NMR ($CDCl_3$, 126 MHz): δ 196.9 (1C), 161.9 (1C), 158.4 (1C), 156.9 (1C), 156.0 (1C), 131.8 (1C), 130.6 (1C), 130.3 (2C), 130.2 (2C), 127.4 (1C), 122.4 (1C), 116.7 (1C), 116.6 (2C+1C, two signals overlapped), 114.4 (2C), 67.2 (2C), 55.5 (1C), 52.4 (2C), 26.6 (1C); IR (KBr, cm^{-1}) 993, 1115, 1165, 1231, 1246, 1267, 1445, 1460, 1493, 1560, 1582, 1599, 1678; HRMS (ESI) *m/z*: [M+Na]$^+$ Calcd for $C_{25}H_{25}NO_4SNa^+$ 458.1396; Found 458.1395.

The regiochemistry was determined by the HMBC experiment.

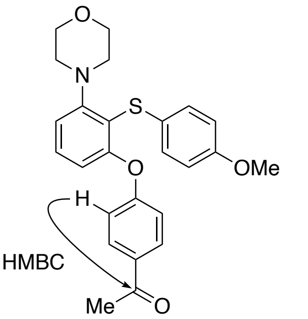

2-(4-Anisylthio)-3-methoxyphenyl *trans*-2-phenylvinyl ether (**8c**)

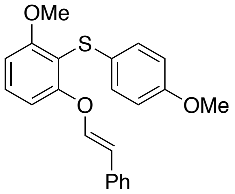

Yield: 44% (8.35 mg, 22.9 μmol); Colorless oil; TLC R_f 0.46 (*n*-hexane/EtOAc = 5/1); ^{1}H NMR ($CDCl_3$, 500 MHz): δ 7.37–7.19 (m, 8H), 7.03 (d, 1H, J = 12.4 Hz), 6.82–6.74 (m, 4H), 6.25 (d, 1H, J = 12.4 Hz), 3.88 (s, 3H), 3.75 (s, 3H); ^{13}C NMR ($CDCl_3$, 126 MHz): δ 161.3 (1C), 159.0 (1C), 158.5 (1C), 143.9 (1C), 135.3 (1C), 131.1 (2C), 130.7 (1C), 128.8 (2C), 127.8 (1C), 126.8 (1C), 125.9 (2C), 114.5 (2C), 114.1 (1C), 113.2 (1C), 110.3 (1C), 106.9 (1C), 56.6 (1C), 55.5 (1C); IR (KBr, cm^{-1}) 772, 1128, 1219, 1240, 1435, 1464, 1493, 1572, 1584, 1655; HRMS (ESI) *m/z*: [M+H]$^+$ Calcd for $C_{22}H_{21}O_3S^+$ 365.1206; Found 365.1197.

The regiochemistry was determined by the HMBC and HSQC experiment.

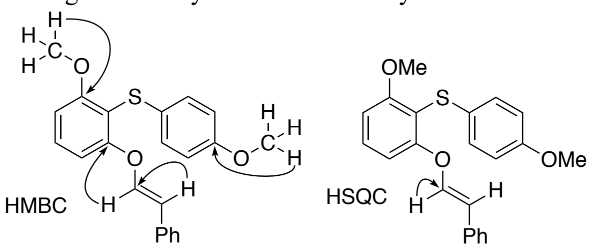

References for Supporting Information

S1 Zhou, C.; Tan, Z.; Jiang, H.; Zhang, M. *Green Chem.* **2018**, *20*, 1992.

S2 Moore, T. L.; O'Connor, D. E. *J. Org. Chem.* **1966**, *31*, 3587.

S3 Xue, F.; Wang, F.; Liu, J.; Di, J.; Liao, Q.; Lu, H.; Zhu, M.; He, L.; He, H.; Zhang, D.; Song, H.; Liu, X.; Qin, Y. *Angew. Chem., Int. Ed.* **2018**, *57*, 6667.

S4 Liu, Q.; Wang, L.; Yue, H.; Li, J.; Luo, Z.; Wei, W. *Green Chem.* **2019**, *21*, 1609.

S5 Maitro, G.; Vogel, S.; Prestat, G.; Madec, D.; Poli, G. *Org. Lett.* **2006**, *8*, 5951.

S6 Xue, Q.; Mao, Z.; Shi, Y.; Mao, H.; Cheng, Y.; Zhu, C. *Tetrahedron Lett.* **2012**, *53*, 1851.

S7 Xu, H.; Lin, Y.; Wan, X.; Yang, C.; Feng, Y. *Tetrahedron Lett*. **2010**, *66*, 8823.

S8 Iriuchijima, S.; Sakakibara, T.; Tsuchihashi, G. *Agr. Biol. Chem.* **1976**, *40*, 1369.

S9 Li, J.; Yang, X.; Wang, S.; Zhang, L.; Zhou, X.; Wang, S.; Ji, S. *Org. Lett.* **2020**, *22*, 4908.

^{1}H and ^{13}C NMR Spectra of Compounds

^{1}H NMR (400 MHz) and ^{13}C NMR (126 MHz) spectra of 4-anisyl 4-(dimethylamino)phenyl sulfoxide (**3g**) ($CDCl_3$)

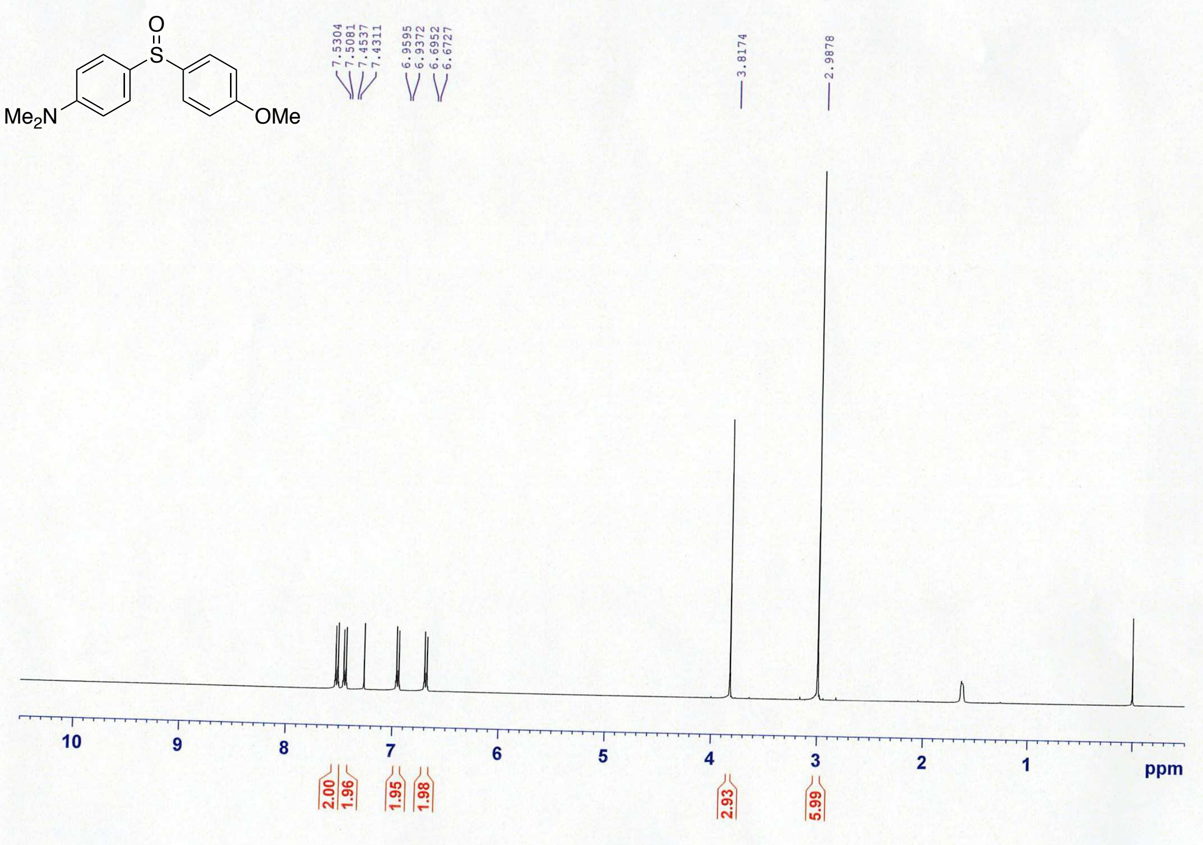

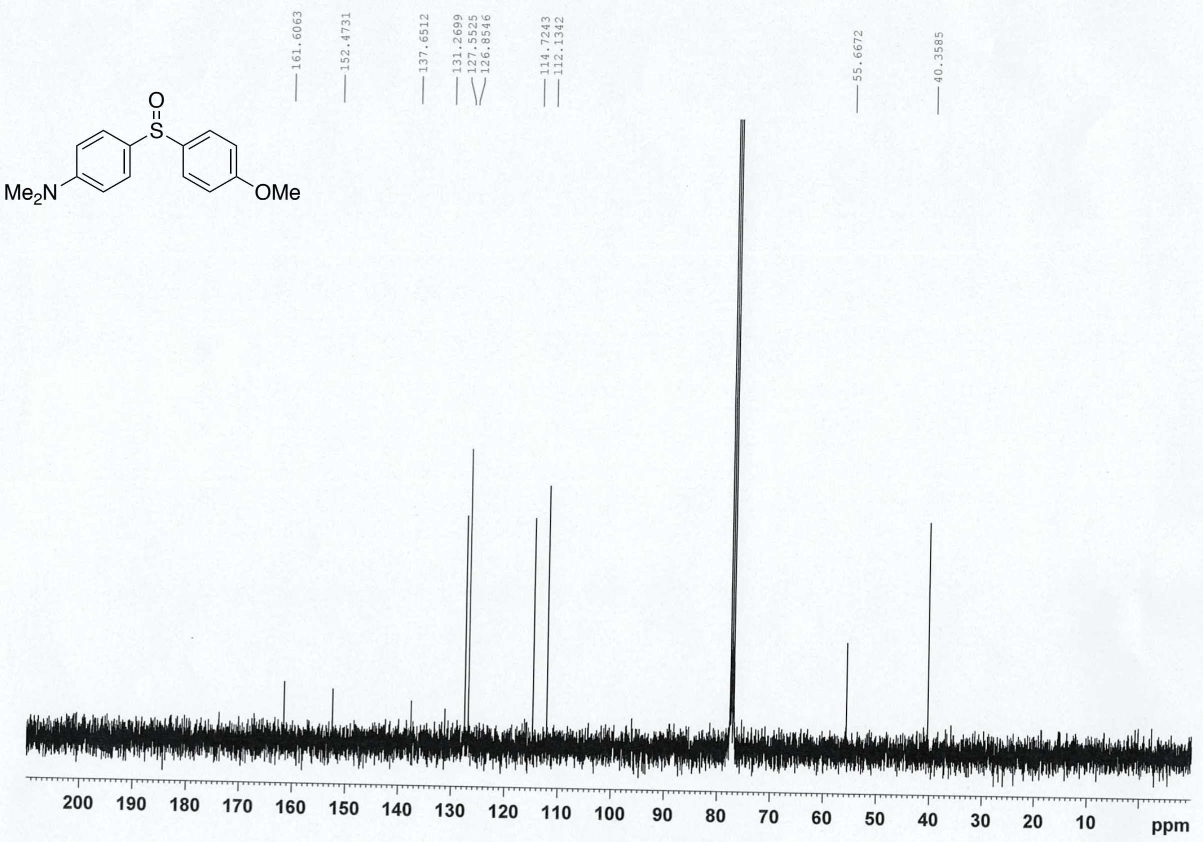

^{1}H NMR (500 MHz) and ^{13}C NMR (126 MHz) spectra of 4-(acetylamino)phenyl 4-anisyl sulfoxide (**3h**) ($CDCl_3$)

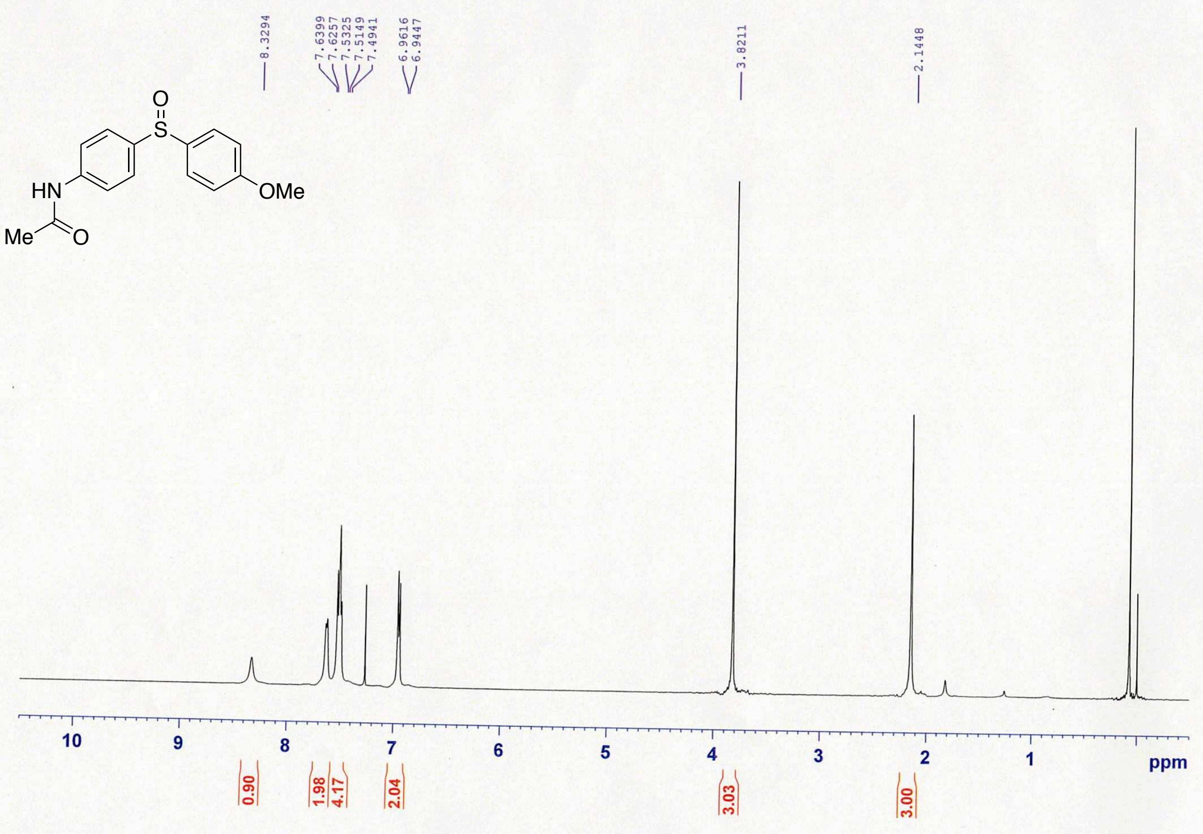

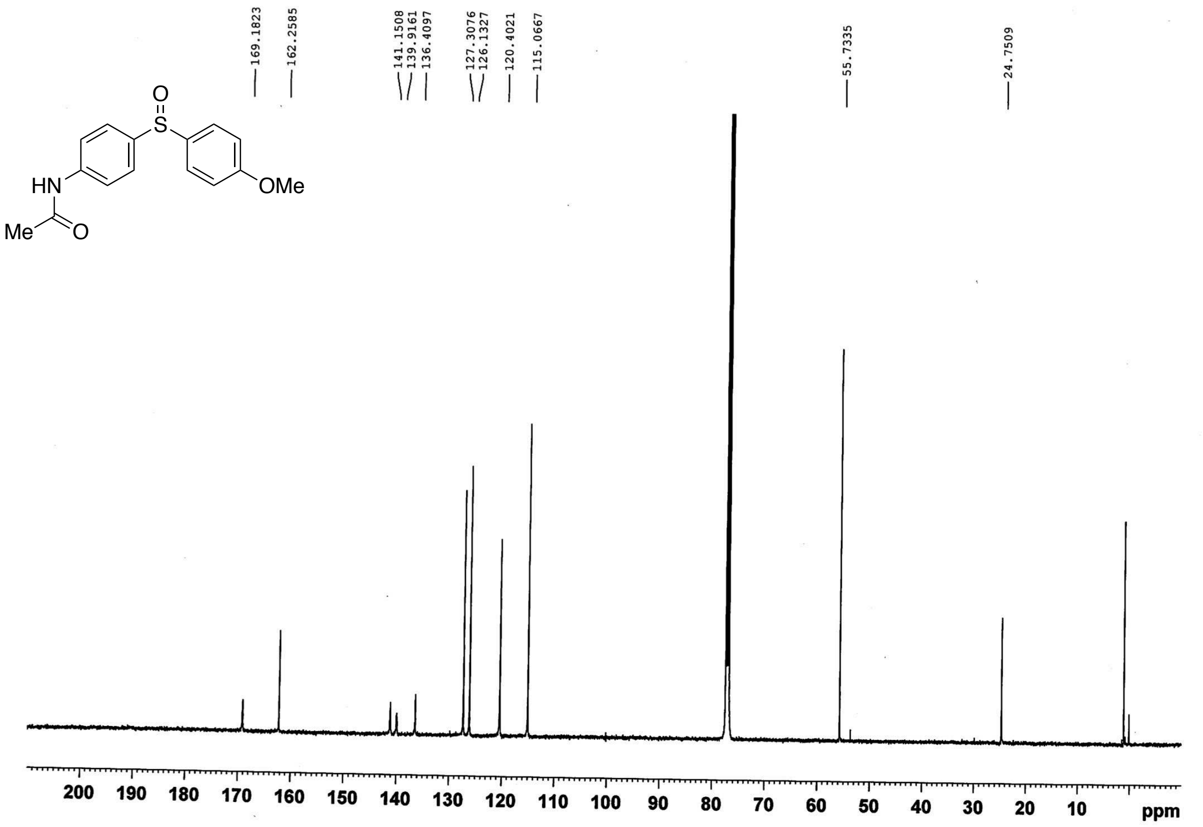

^{1}H NMR (500 MHz) and ^{13}C NMR (126 MHz) spectra of 4-acetylphenyl 4-anisyl sulfoxide (**3j**) ($CDCl_3$)

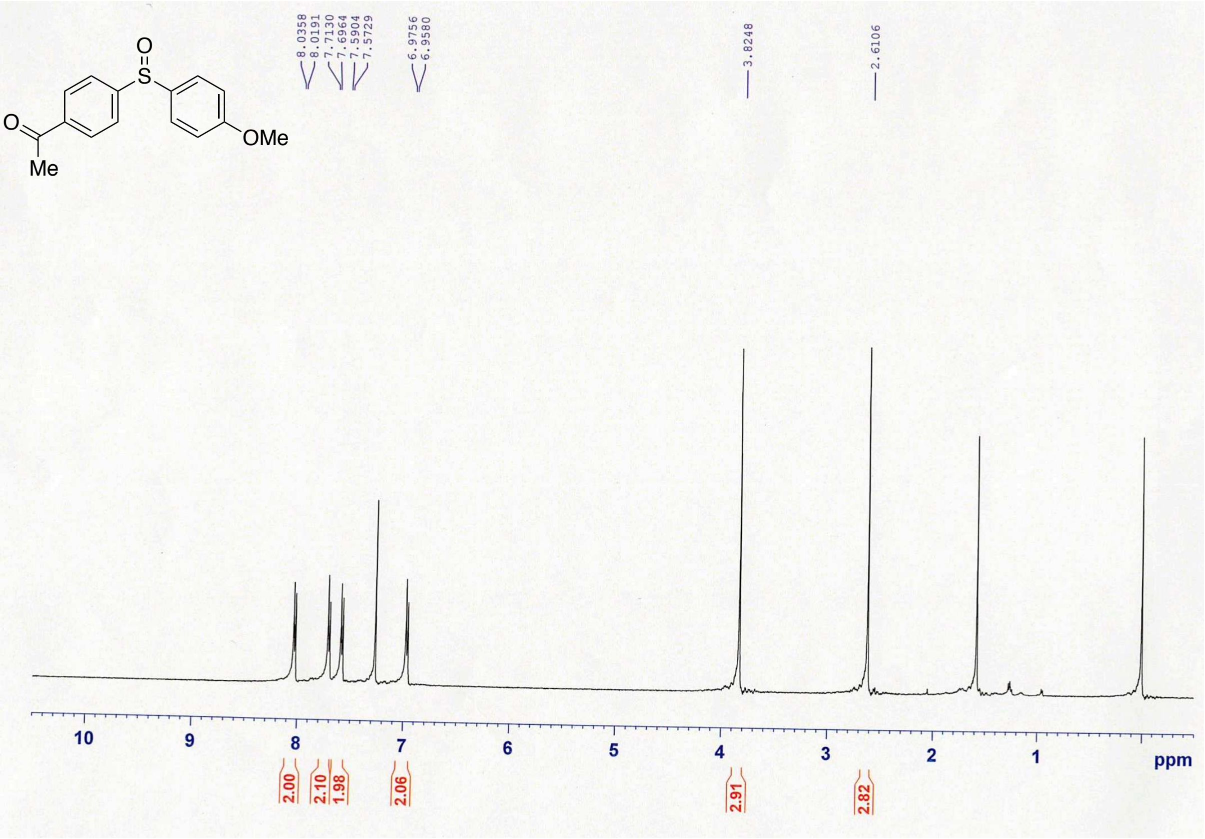

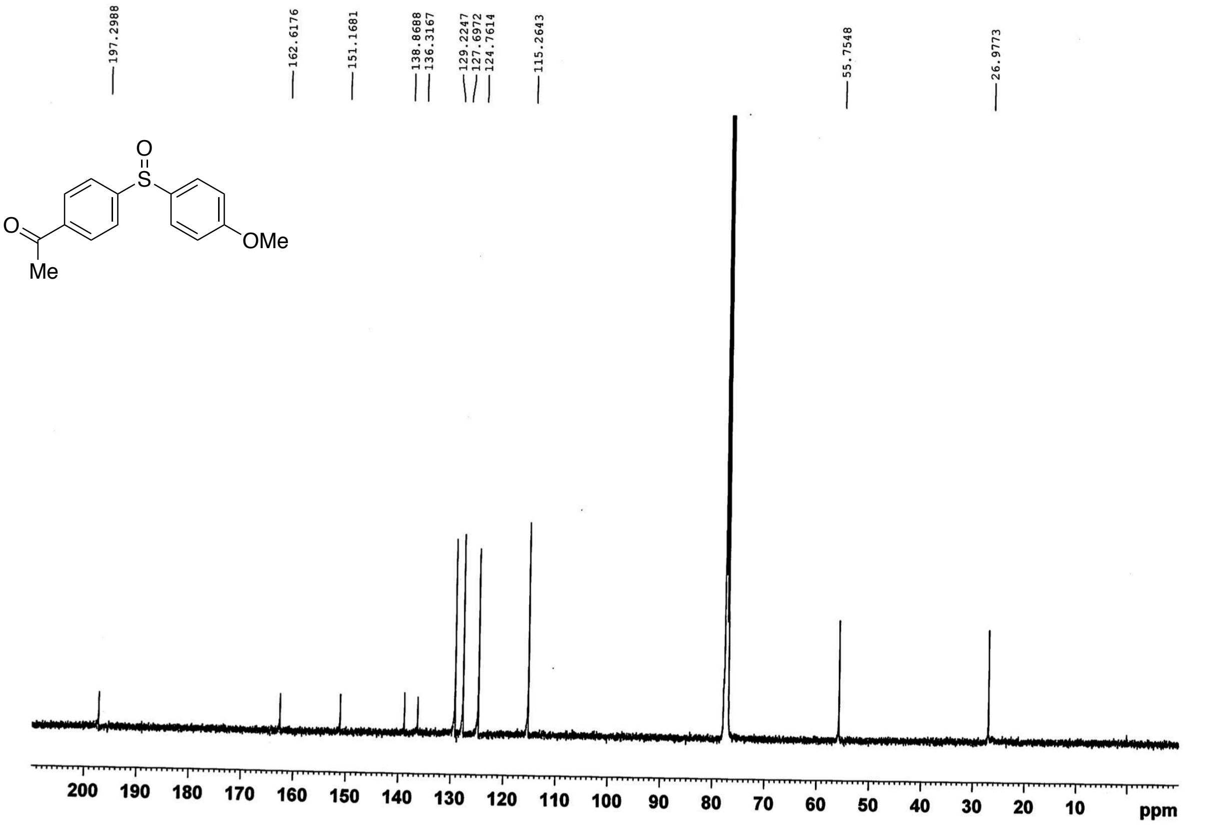

^{1}H NMR (400 MHz) and ^{13}C NMR (126 MHz) spectra of 4-Anisyl 4-vinylphenyl sulfoxide (**3k**) ($CDCl_3$)

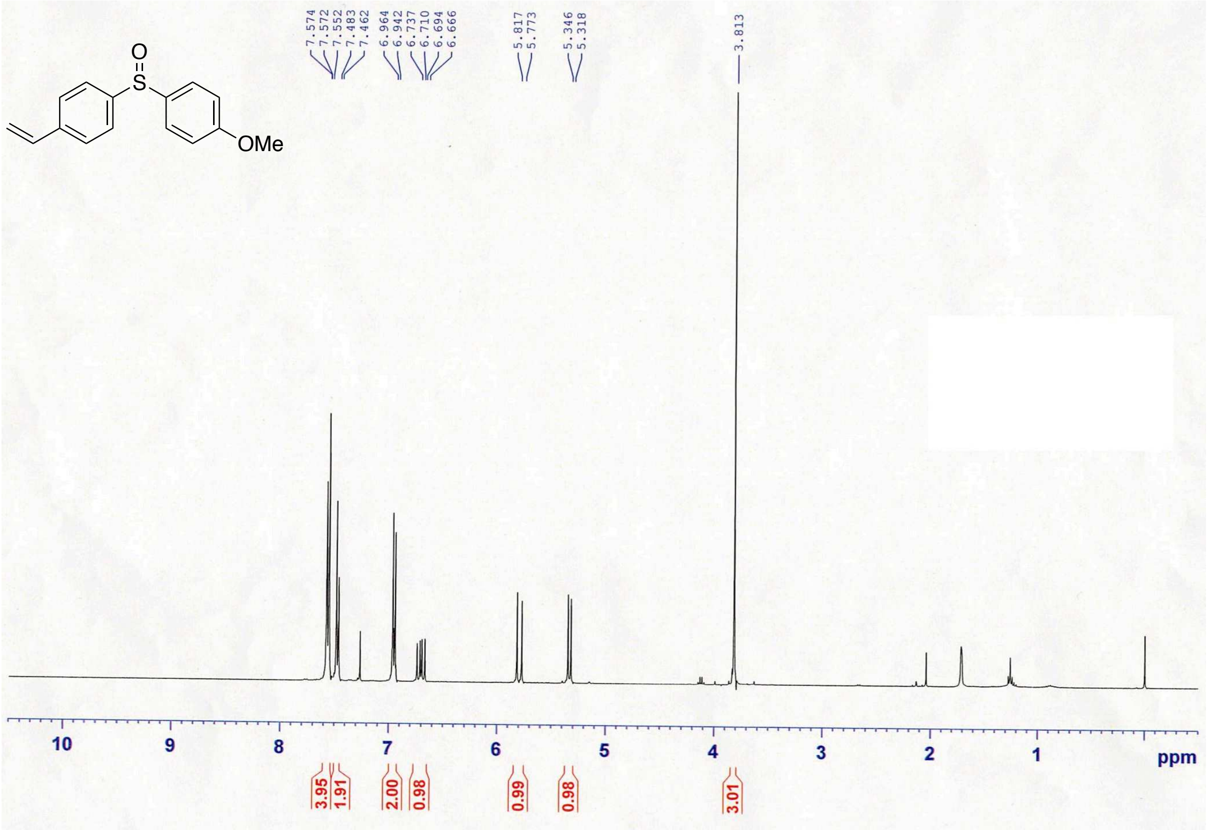

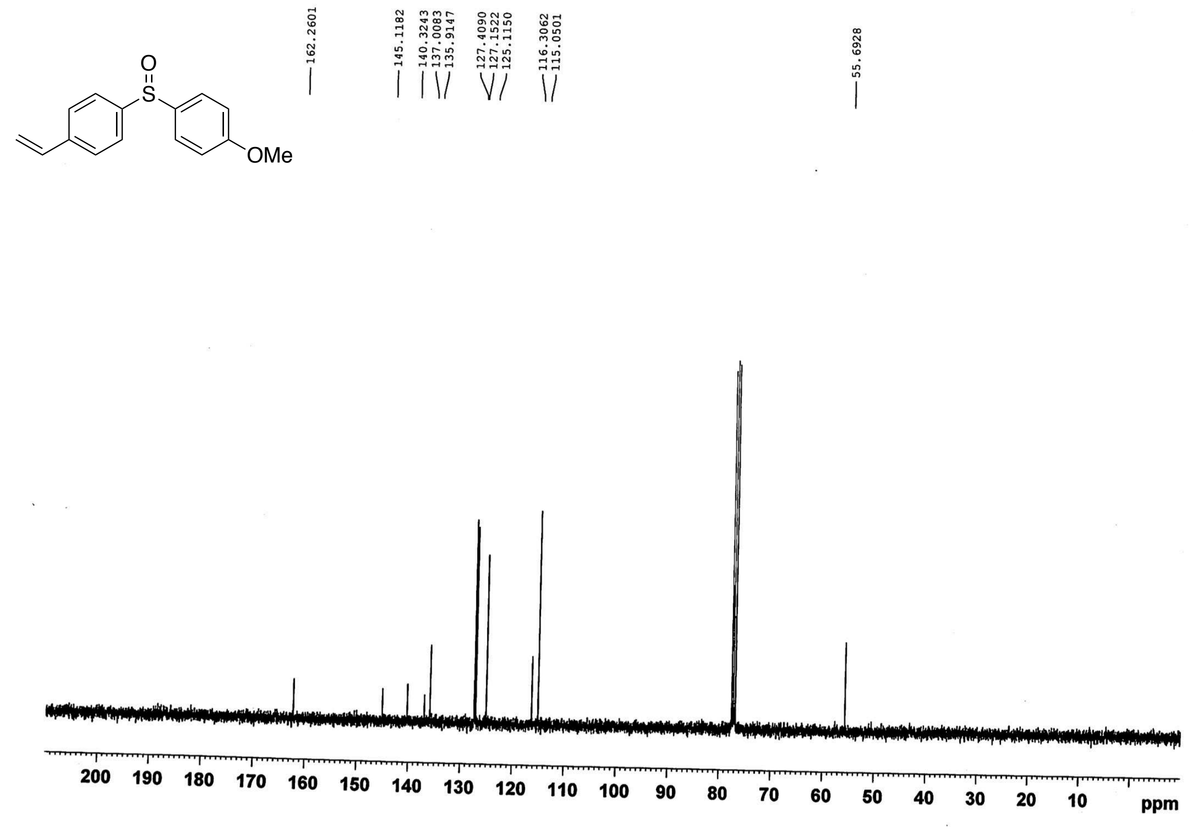

^{1}H NMR (400 MHz) and ^{13}C NMR (126 MHz) spectra of 4-anisyl 4-(methylthio)phenyl sulfoxide (**3l**) ($CDCl_3$)

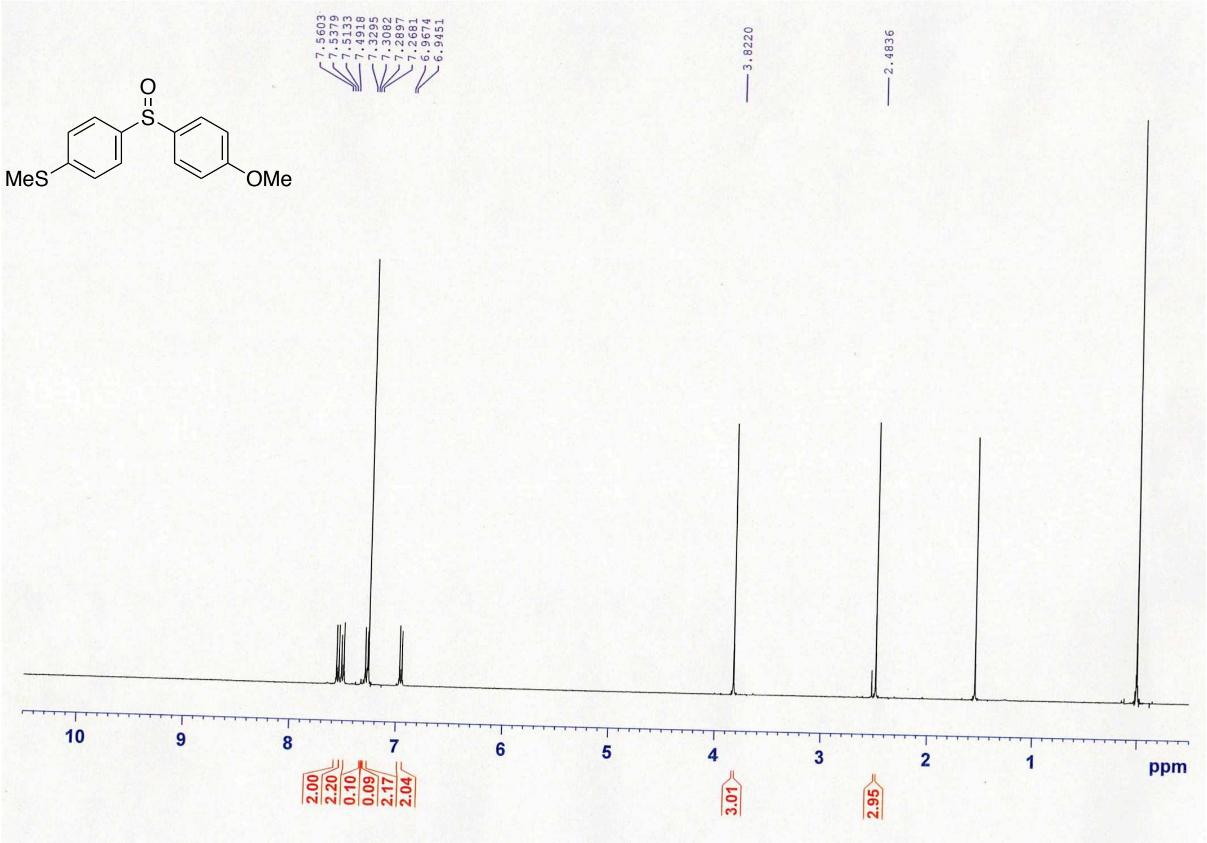

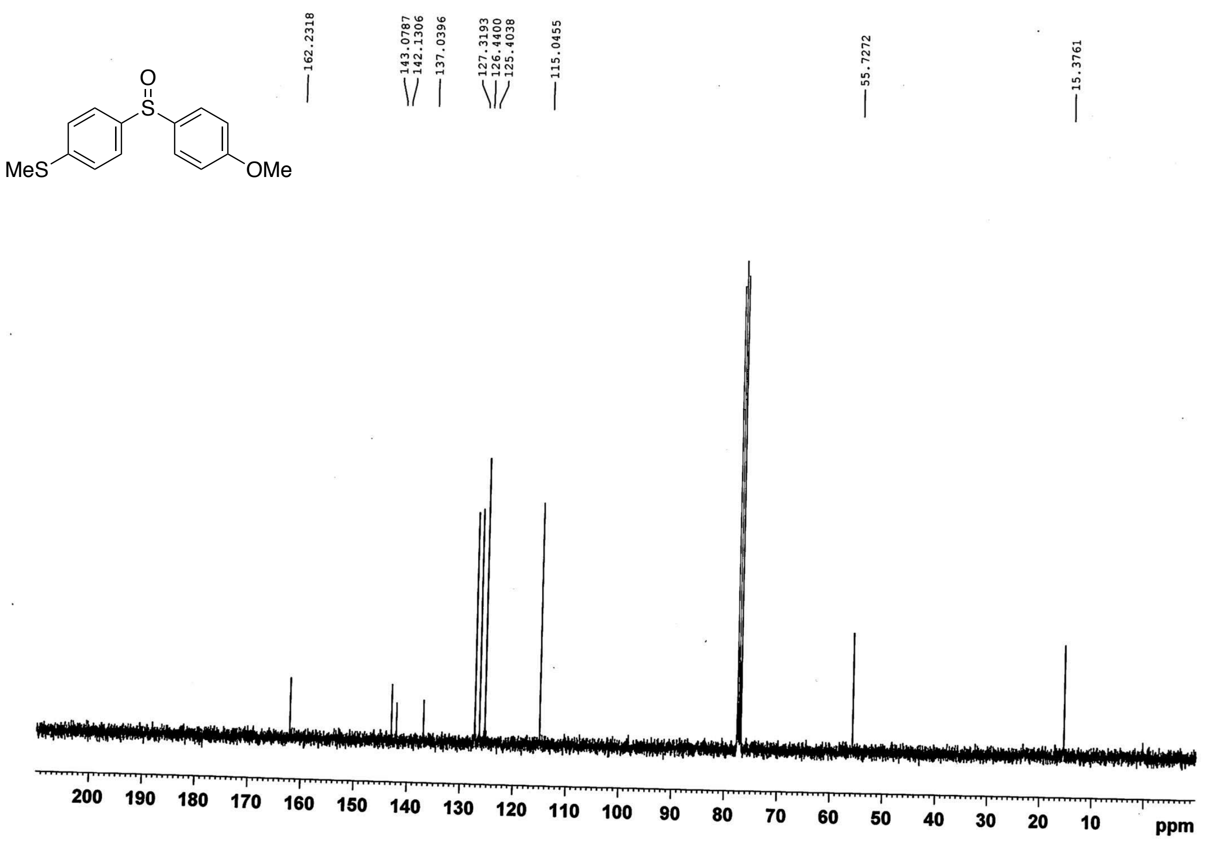

^{1}H NMR (500 MHz) and ^{13}C NMR (126 MHz) spectra of *trans*-2-cyclohexylvinyl 4-methoxyphenyl sulfoxide (**3n**) ($CDCl_3$)

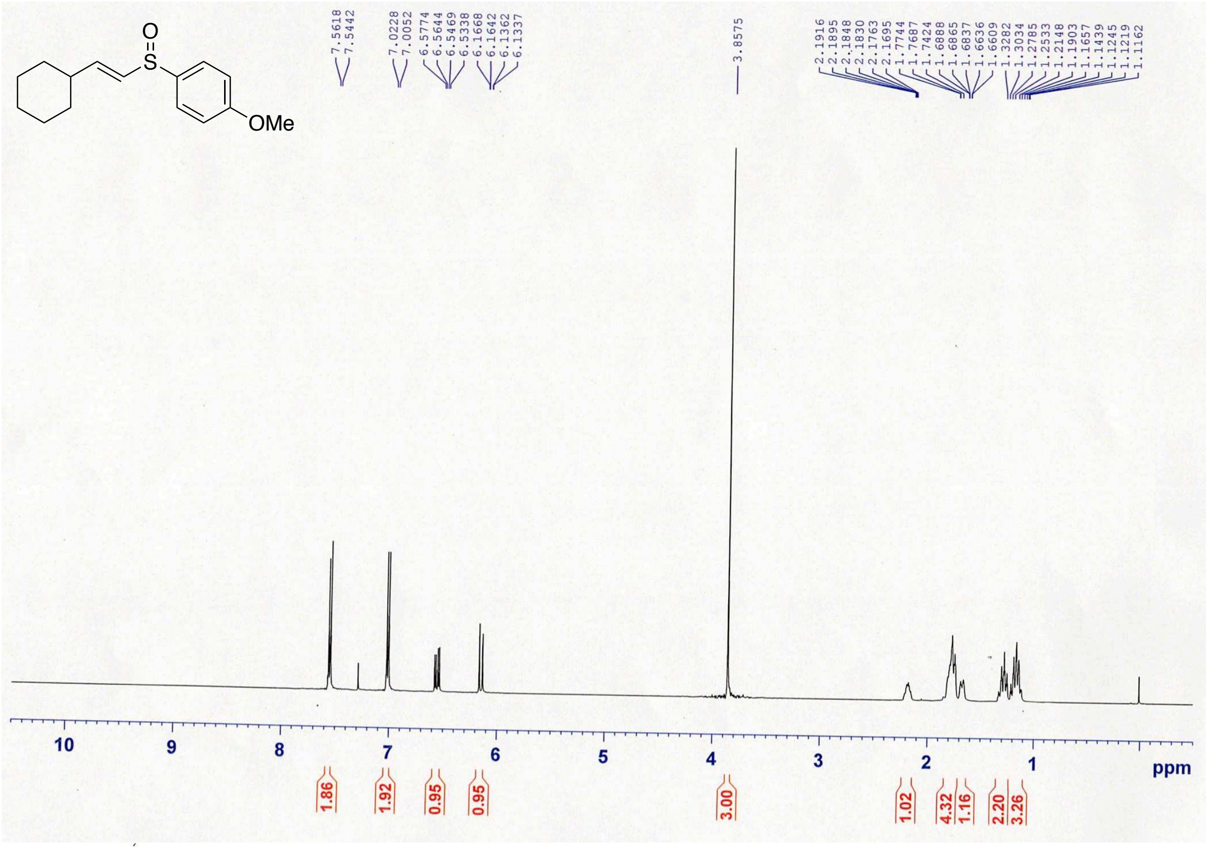

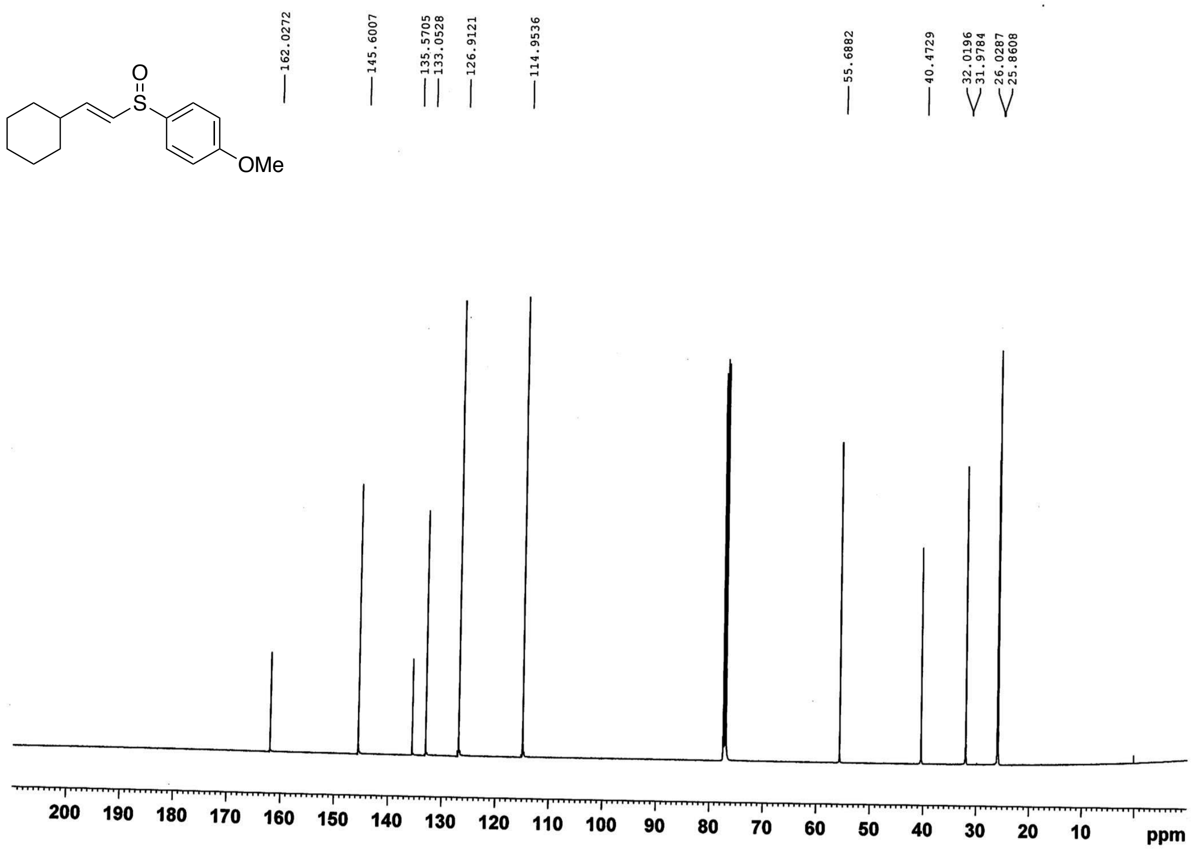

^{1}H NMR (500 MHz) and ^{13}C NMR (126 MHz) spectra of 2-(*tert*-butoxycarbonylamino)ethyl 4-tolyl sulfoxide (**3v**) ($CDCl_3$)

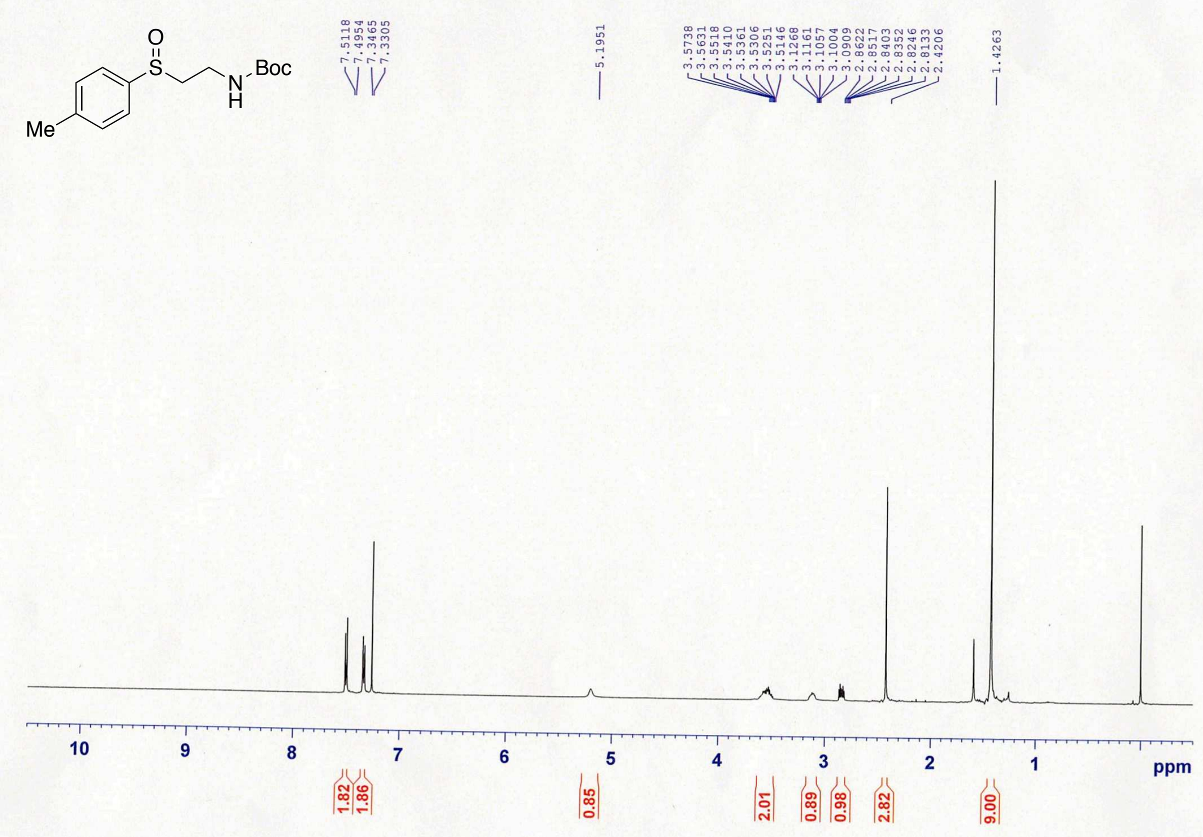

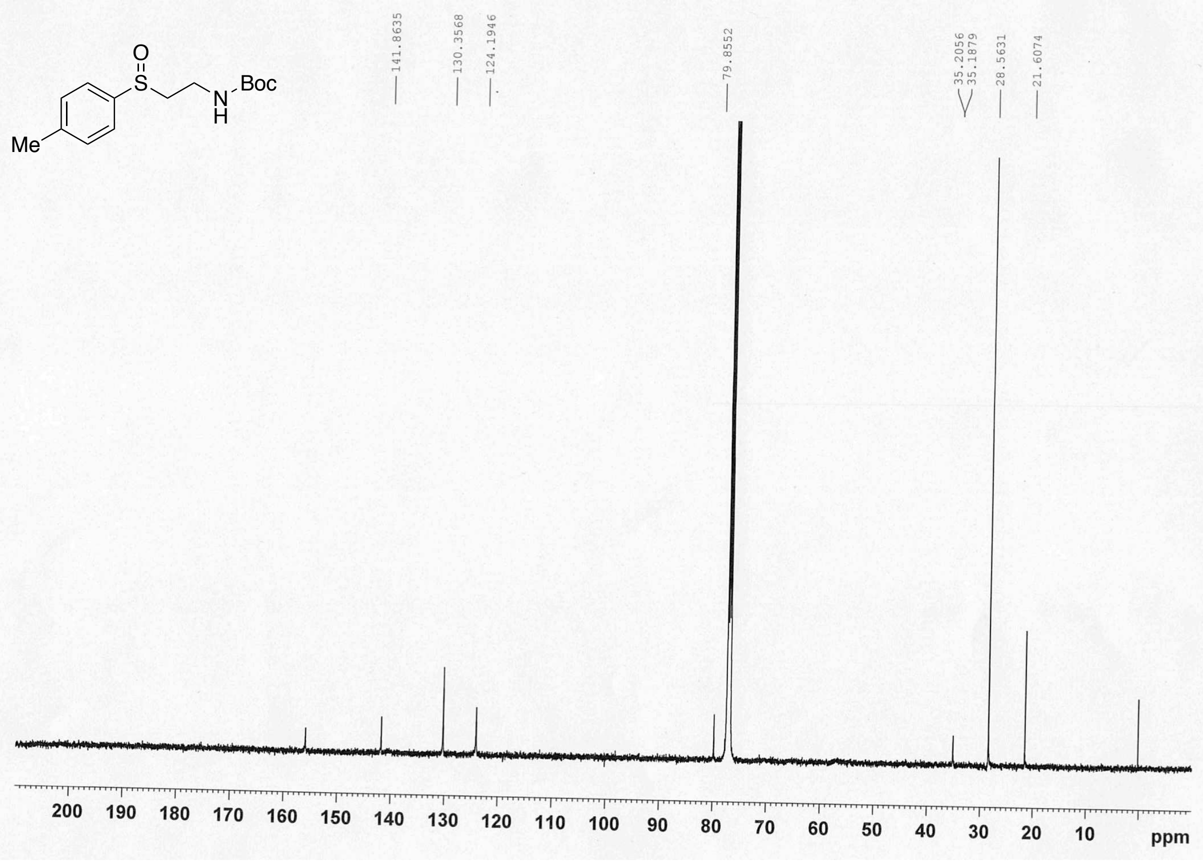

^{1}H NMR (500 MHz) and ^{13}C NMR (126 MHz) spectra of methyl 4-(4-tolyl)benzenesulfinate (**5a**) ($CDCl_3$)

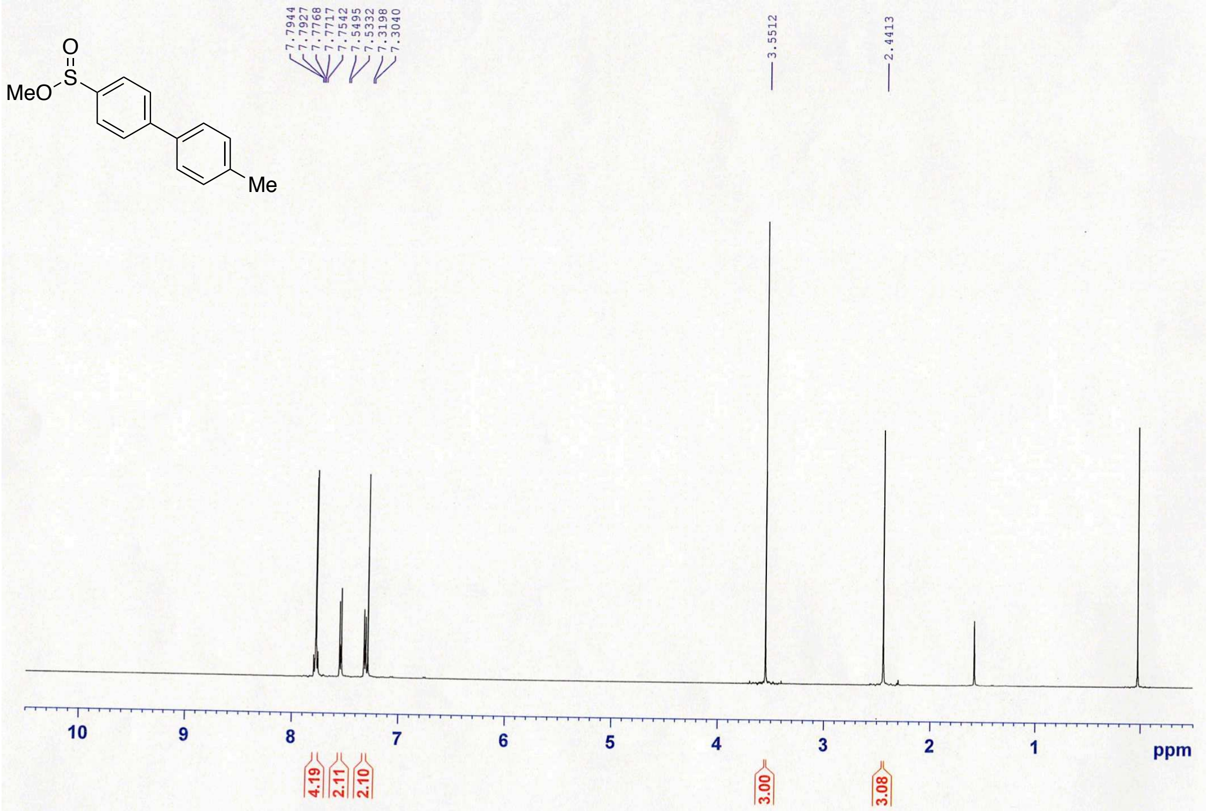

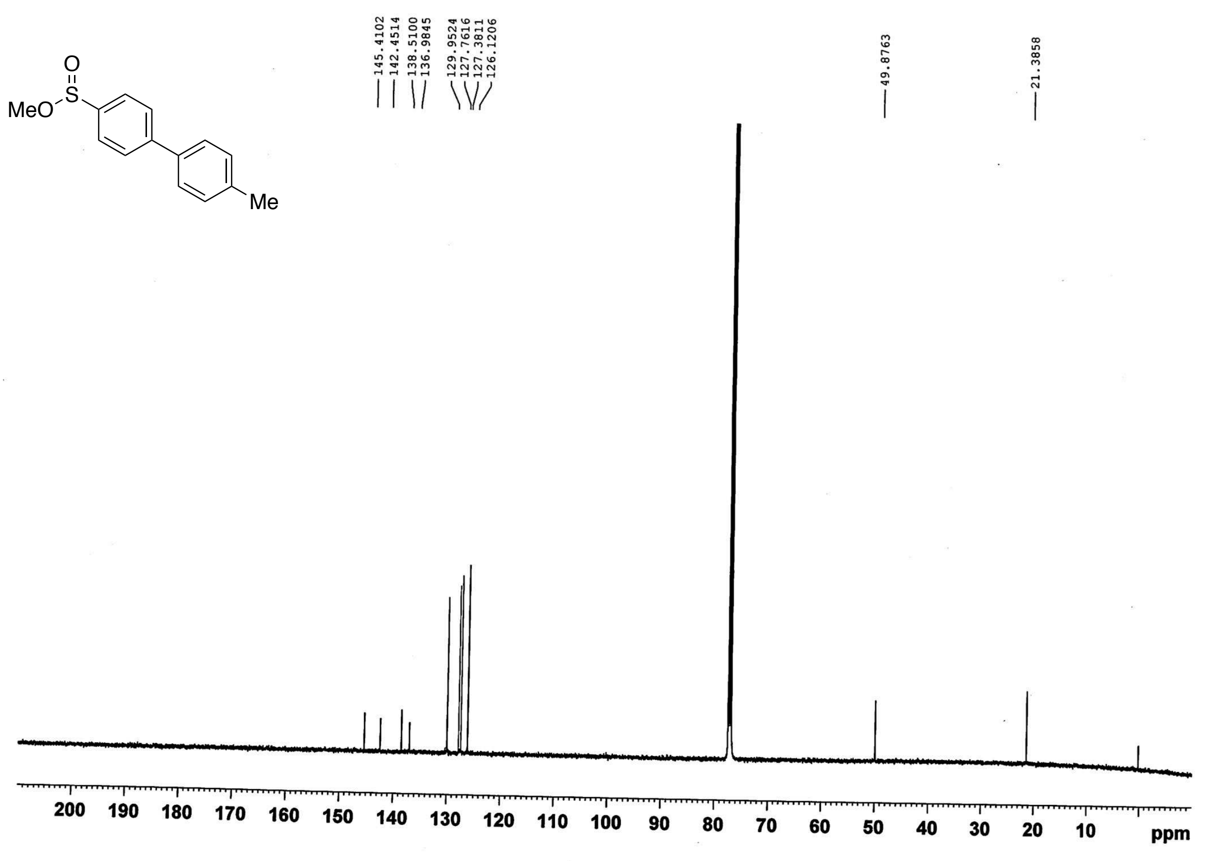

^{1}H NMR (500 MHz) and ^{13}C NMR (126 MHz) spectra of methyl 4-(4-formylphenyl)benzenesulfinate (**5b**) ($CDCl_3$)

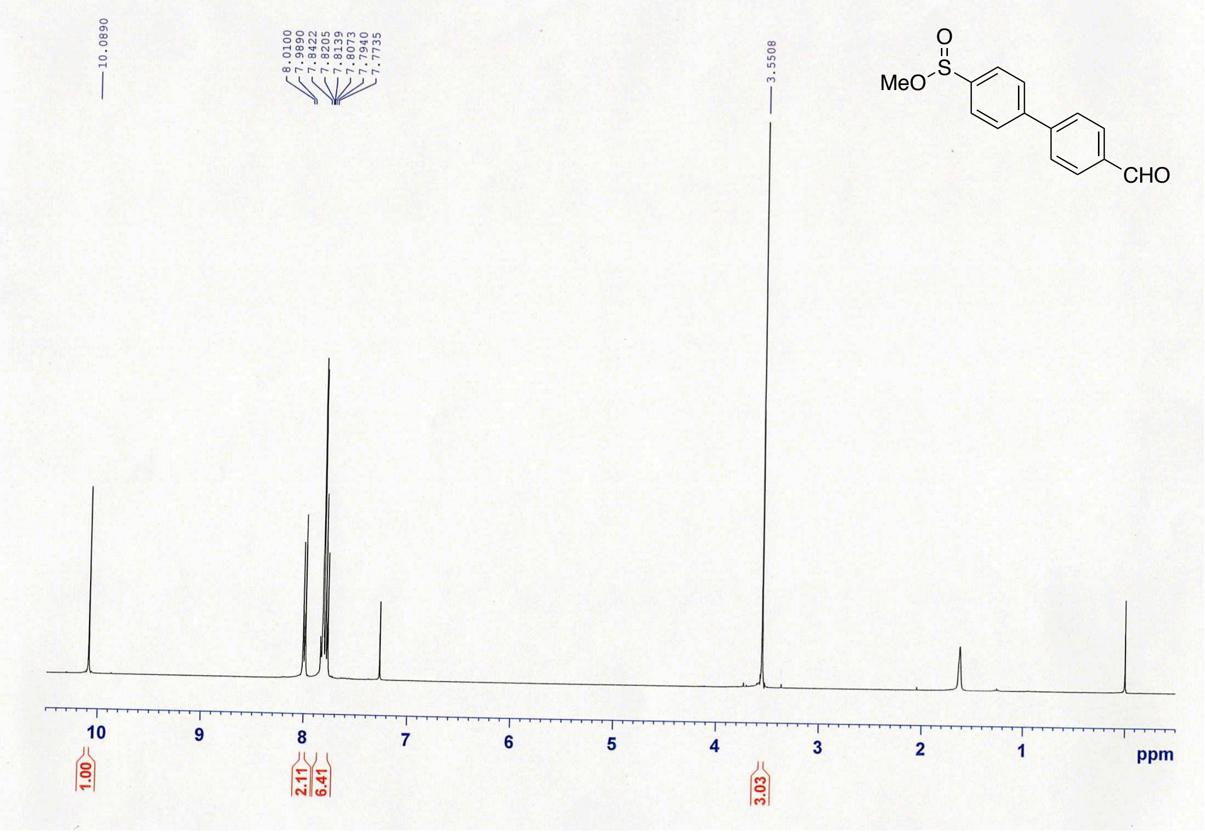

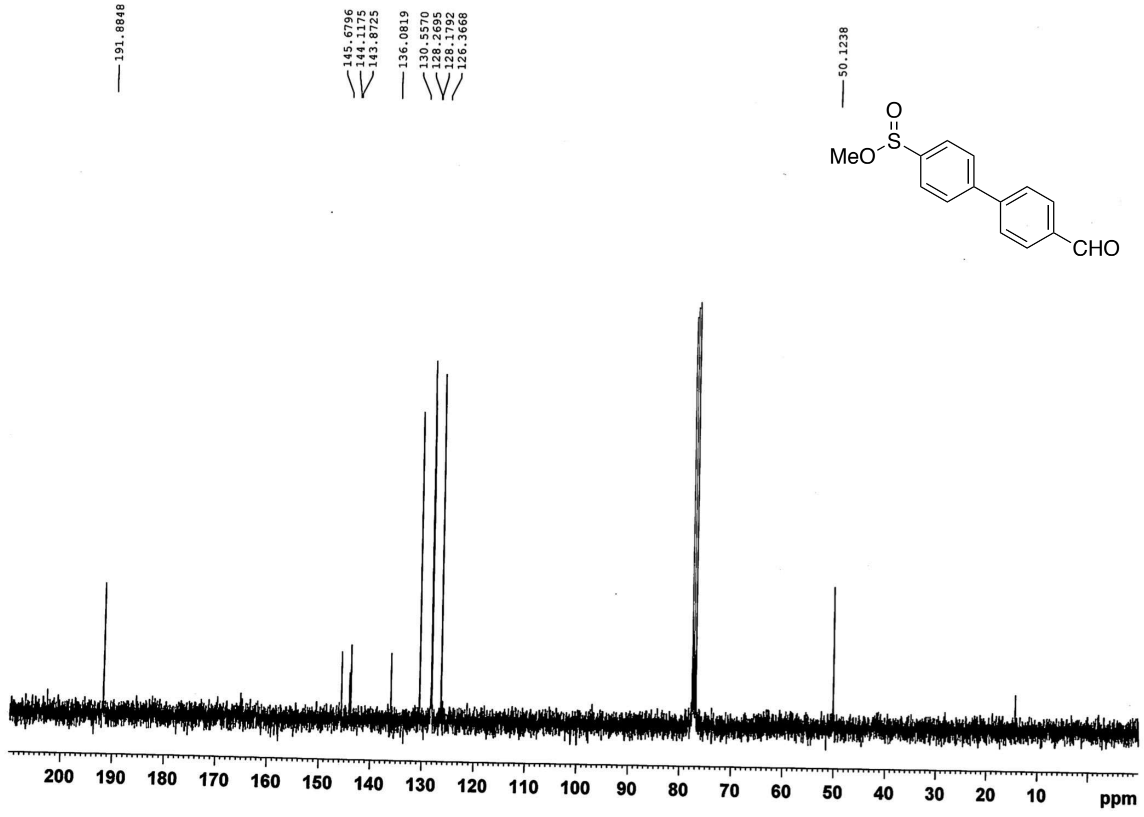

^{1}H NMR (500 MHz) and ^{13}C NMR (126 MHz) spectra of methyl 4-(4-(acetylamino)phenyl)benzenesulfinate (**5c**) ($CDCl_3$)

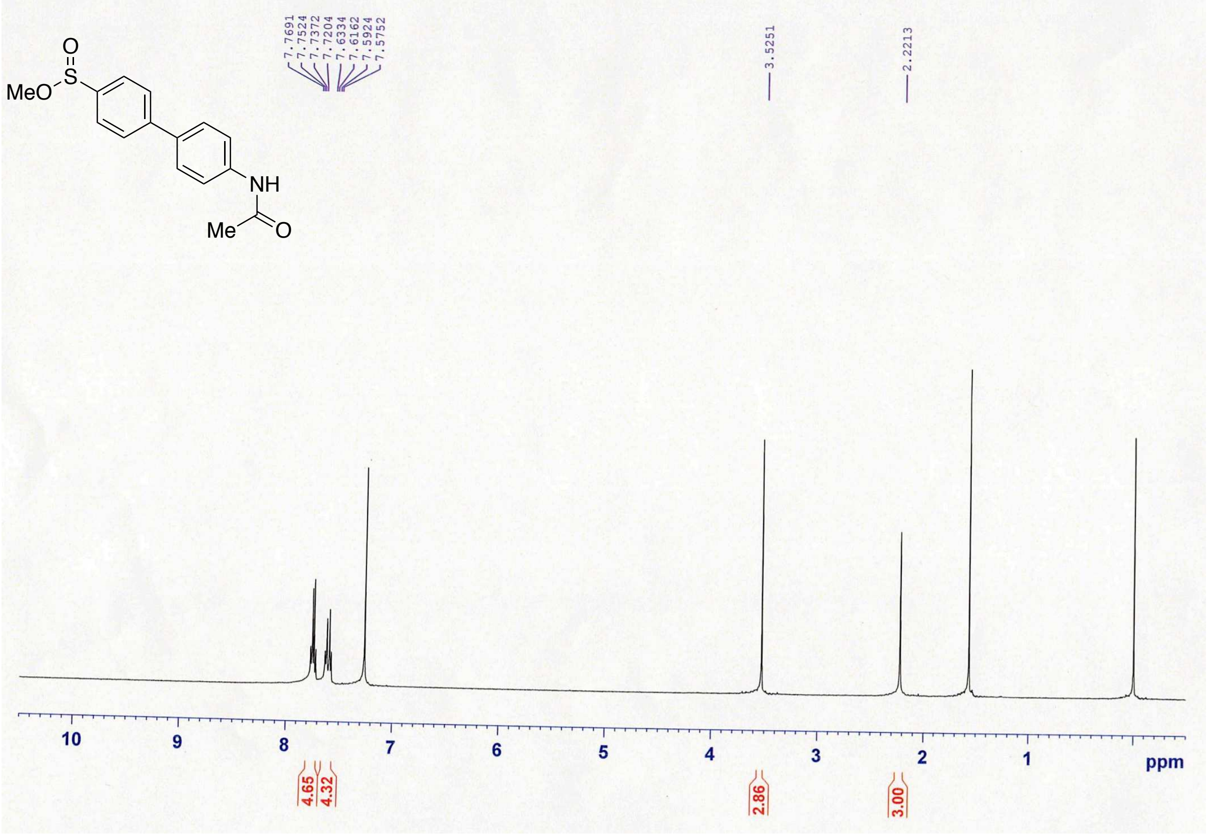

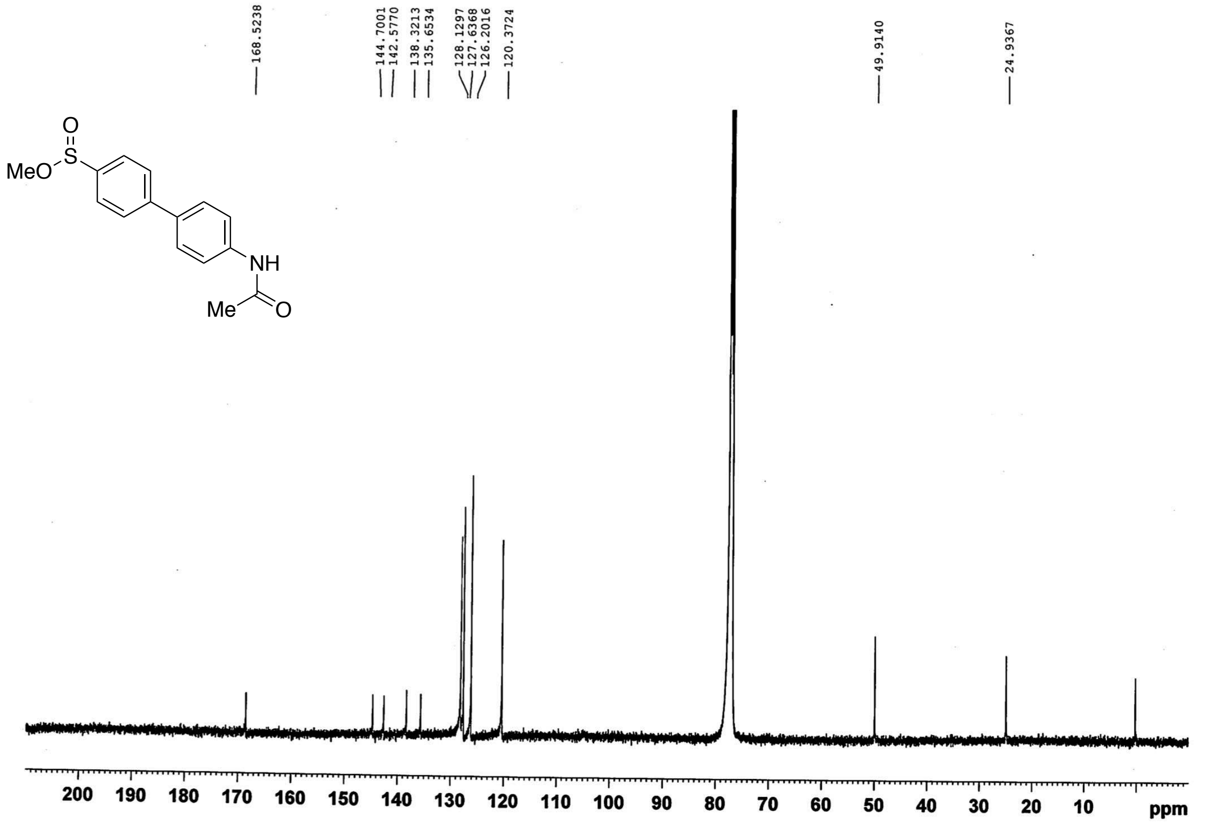

^{1}H NMR (500 MHz) and ^{13}C NMR (126 MHz) spectra of methyl 4-(4-vinylphenyl)benzenesulfinate (**5d**) ($CDCl_3$)

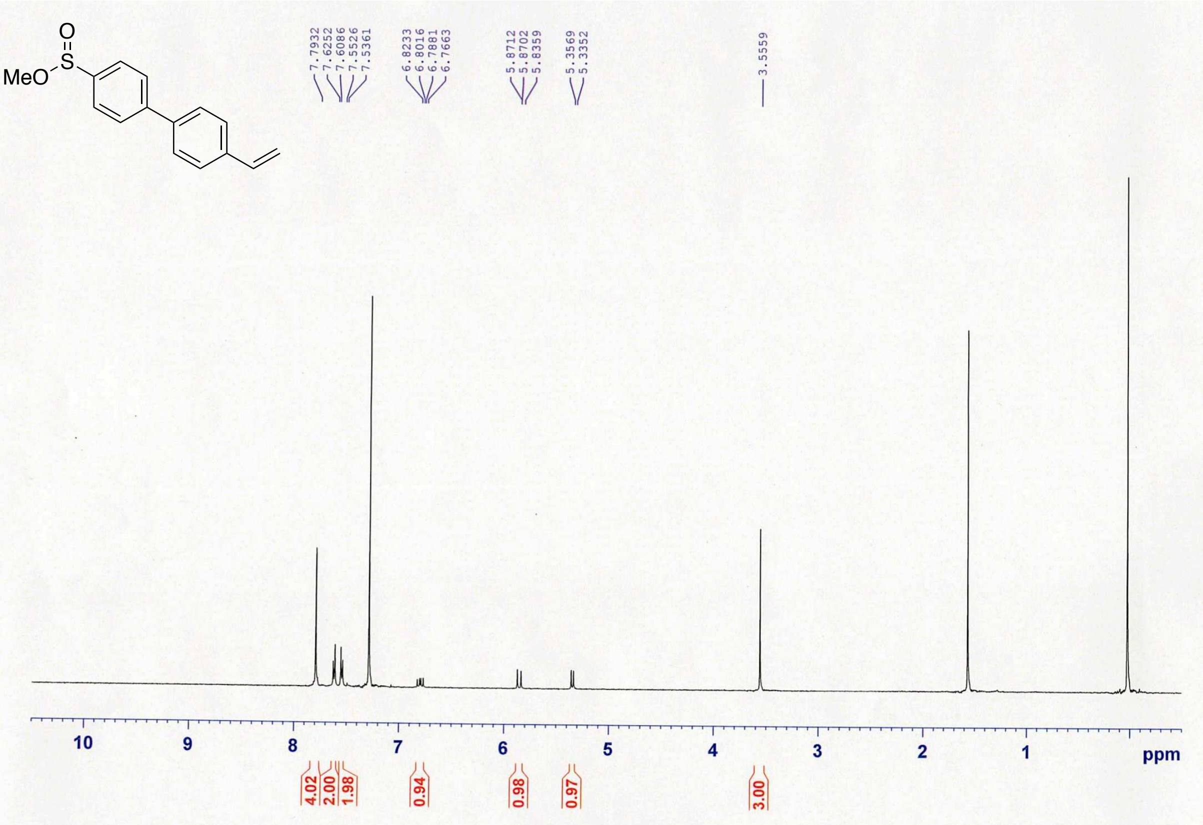

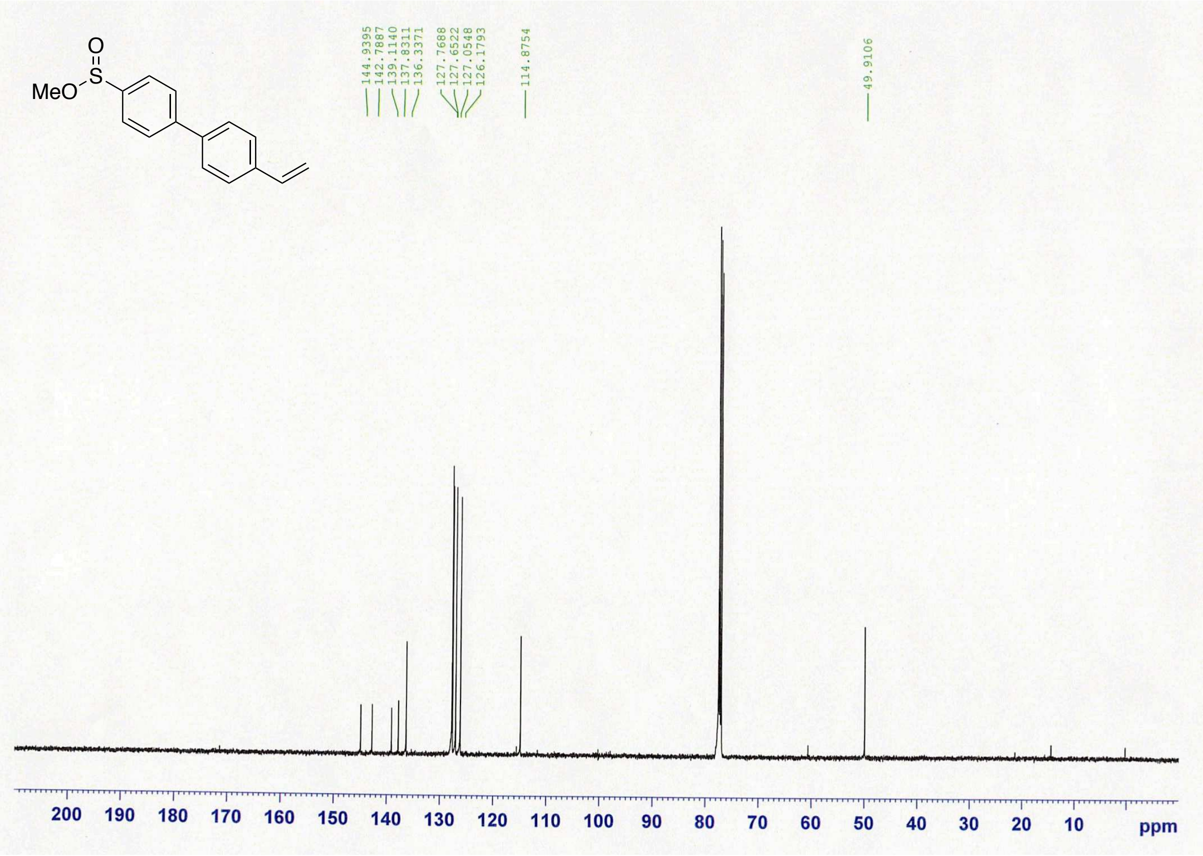

^{1}H NMR (500 MHz) and ^{13}C NMR (126 MHz) spectra of 4-hydroxyphenyl 4-(4-tolyl)phenyl sulfoxide (**6a**) ($CDCl_3$)

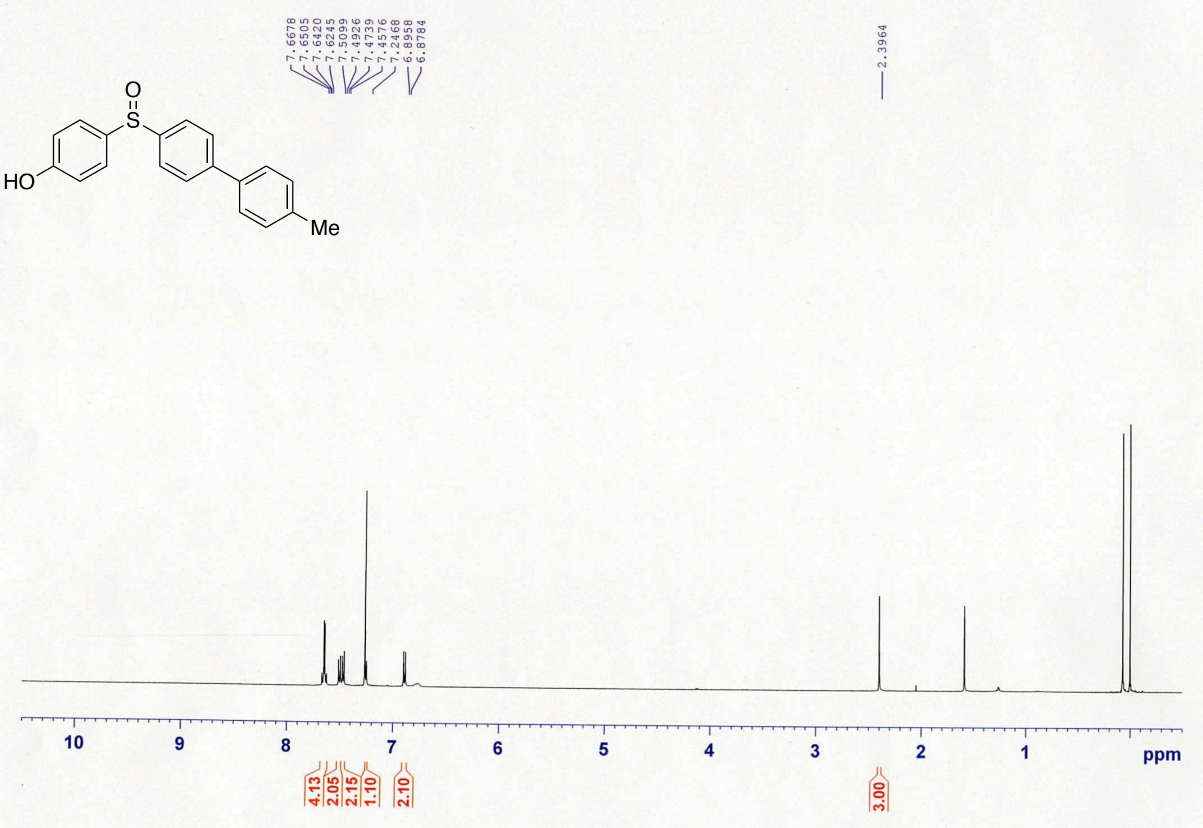

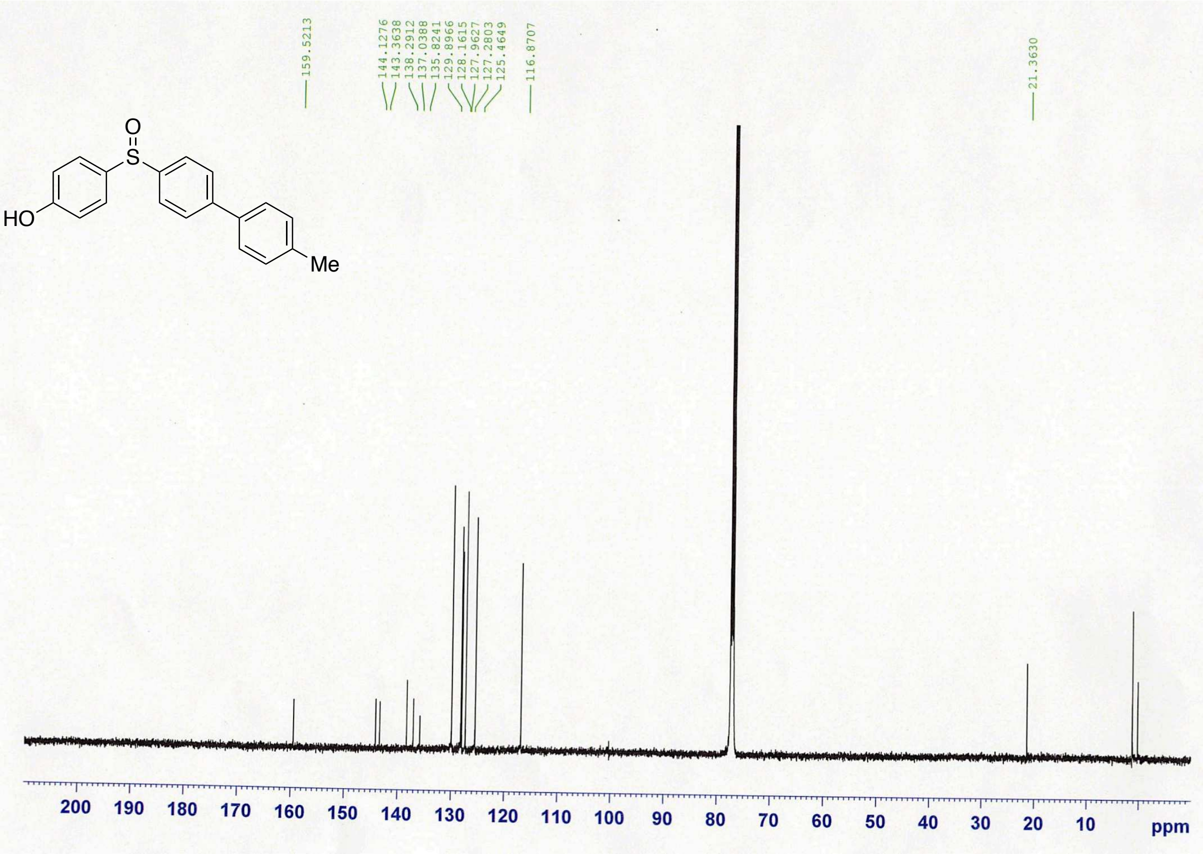

^{1}H NMR (500 MHz) and ^{13}C NMR (126 MHz) spectra of 4-(dimethylamino)phenyl 4-(4-formylphenyl)phenyl sulfoxide (**6b**) ($CDCl_3$)

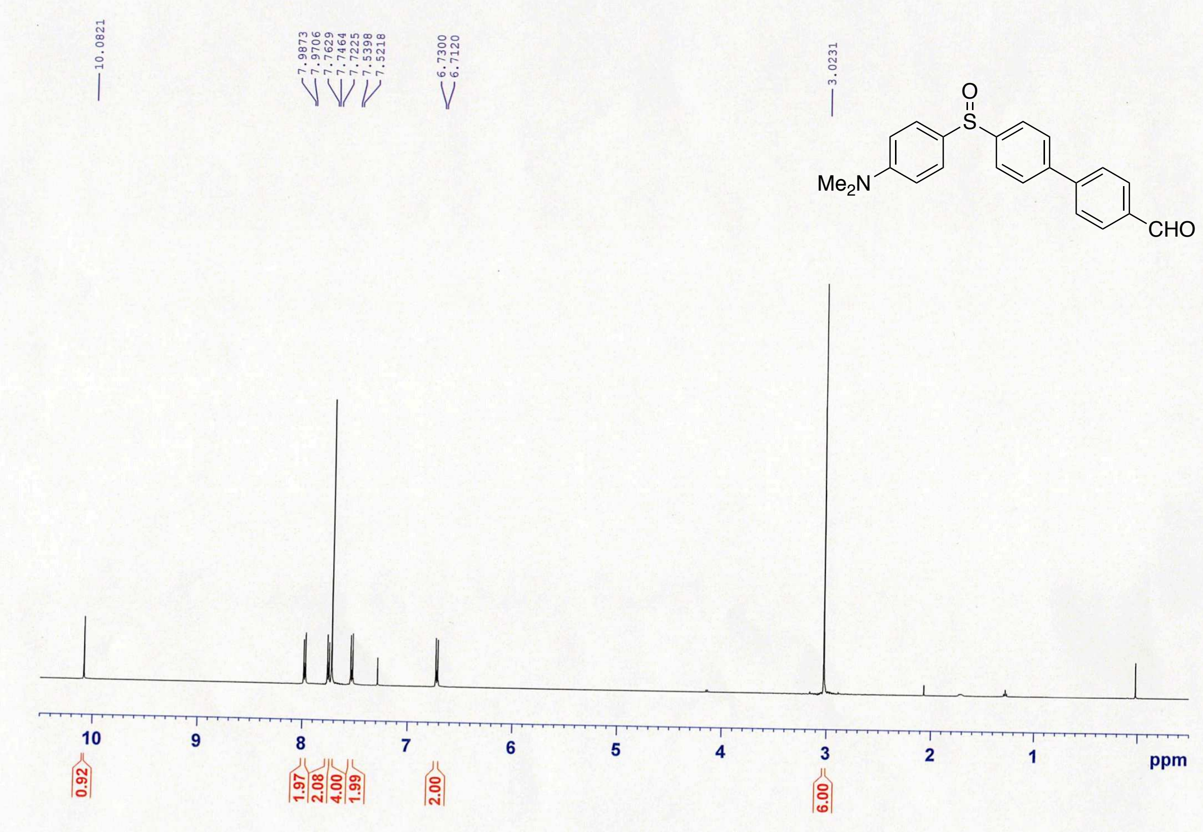

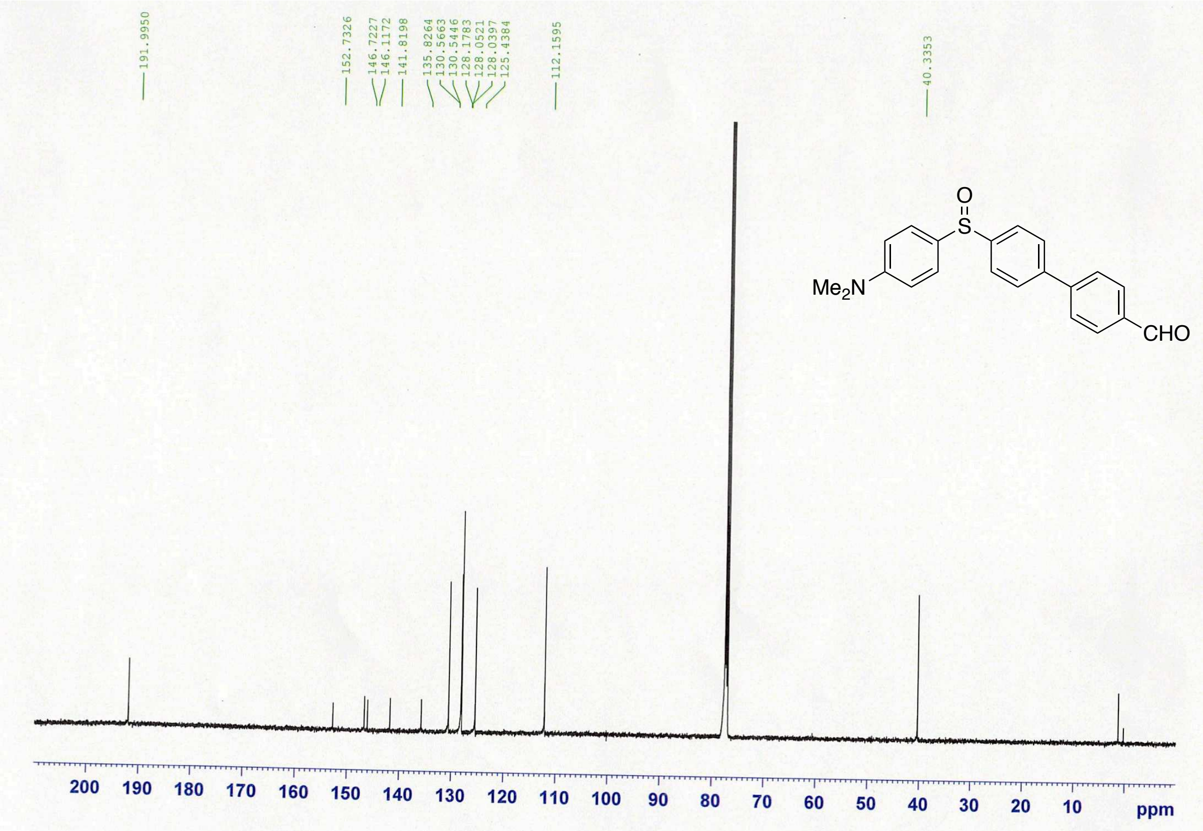

^{1}H NMR (400 MHz) and ^{13}C NMR (126 MHz) spectra of 4-(4-(acetylamino)phenyl) 4-(methylthio)phenyl sulfoxide (**6c**) ($CDCl_3$)

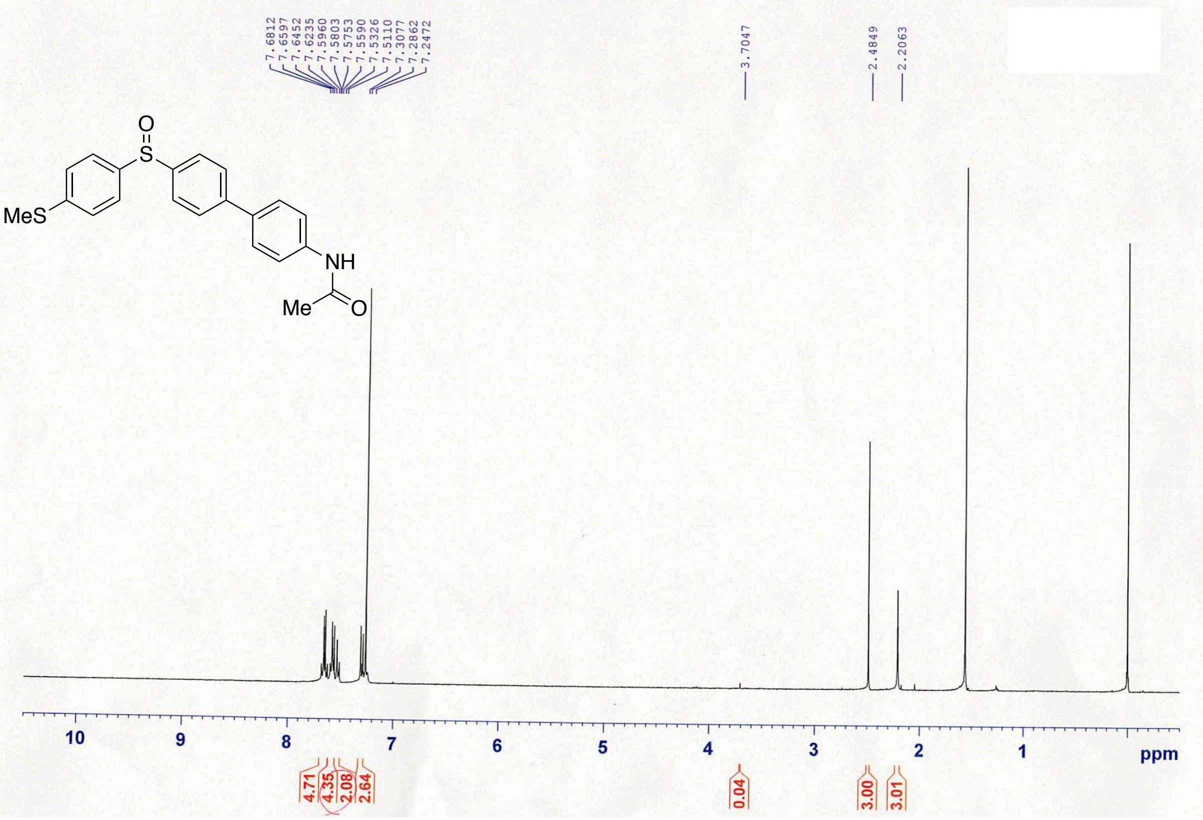

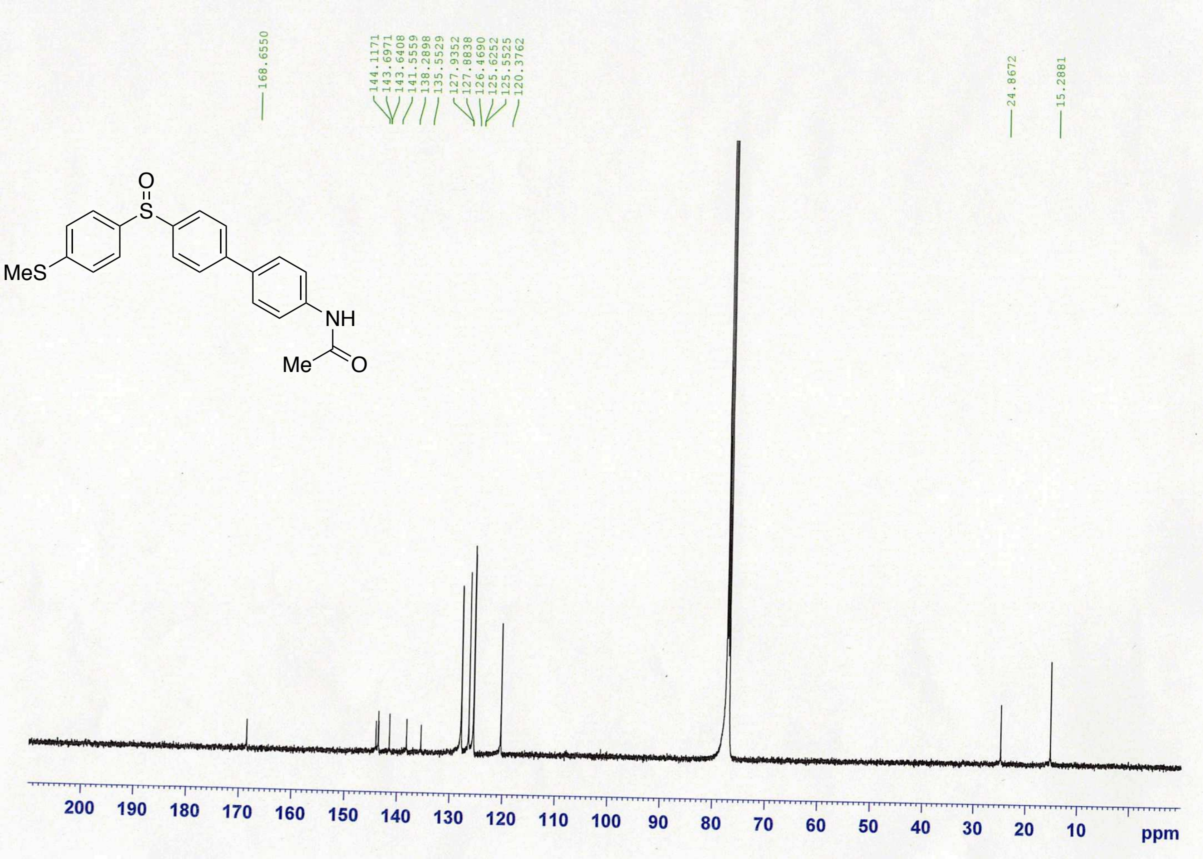

^{1}H NMR (500 MHz) and ^{13}C NMR (126 MHz) spectra of 4-(methylthio)phenyl 4-(4-vinylphenyl)phenyl sulfoxide (**6d**) ($CDCl_3$)

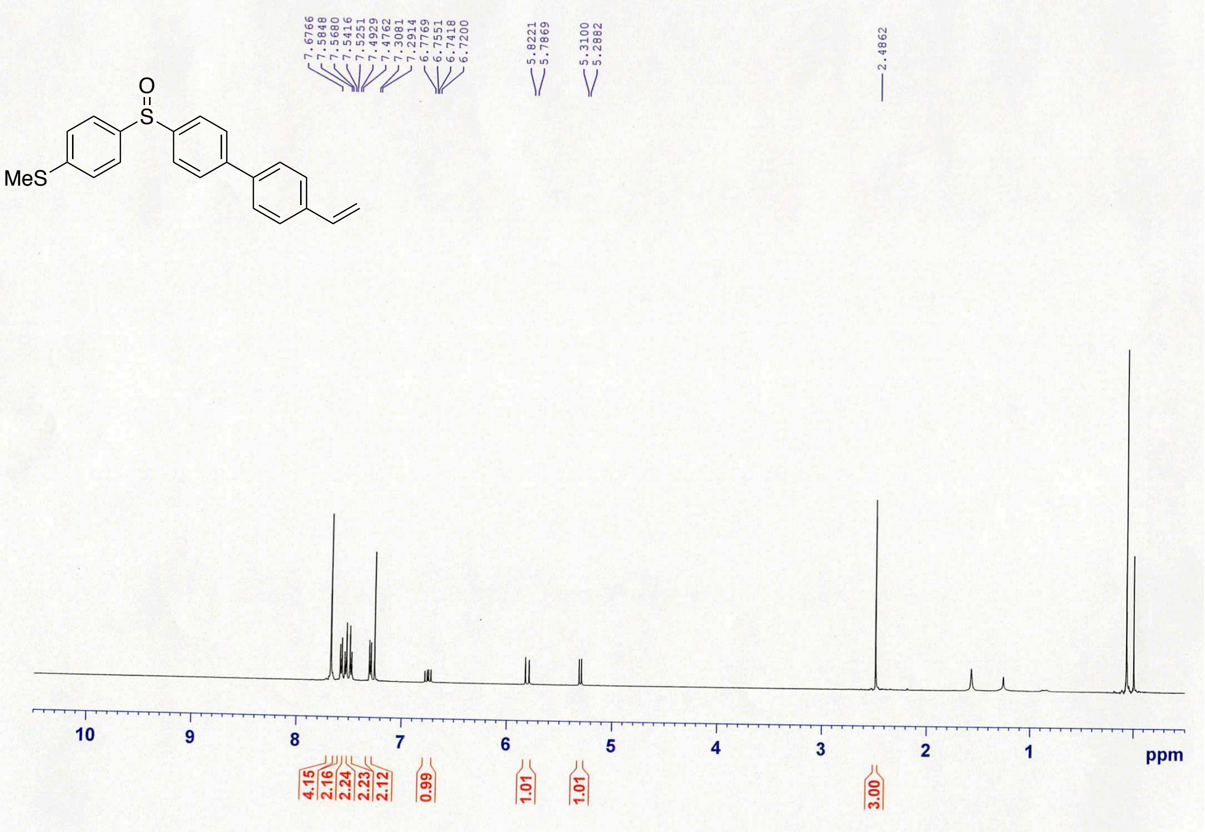

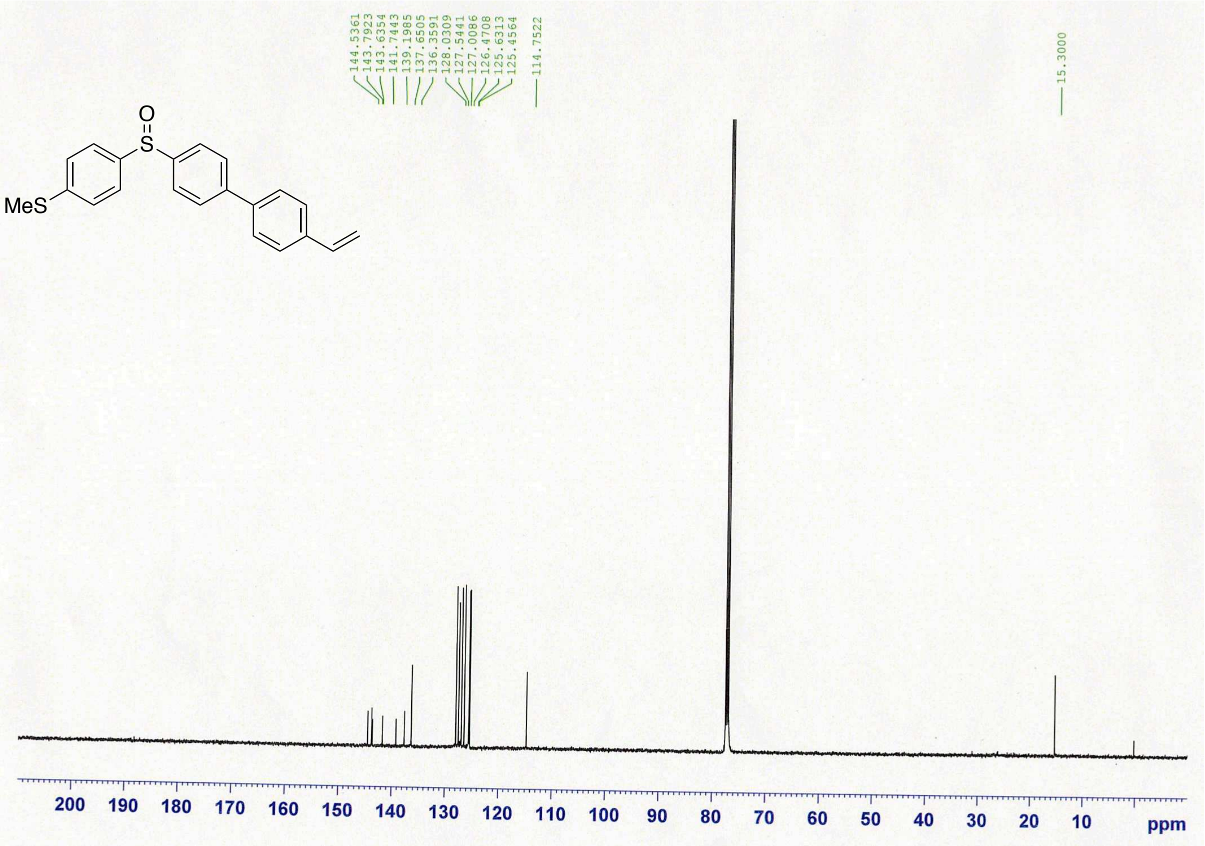

^{1}H NMR (400 MHz) and ^{13}C NMR (126 MHz) spectra of 4-acetylphenyl 2-(4-anisylthio)-3-methoxyphenyl ether (**8a**) ($CDCl_3$)

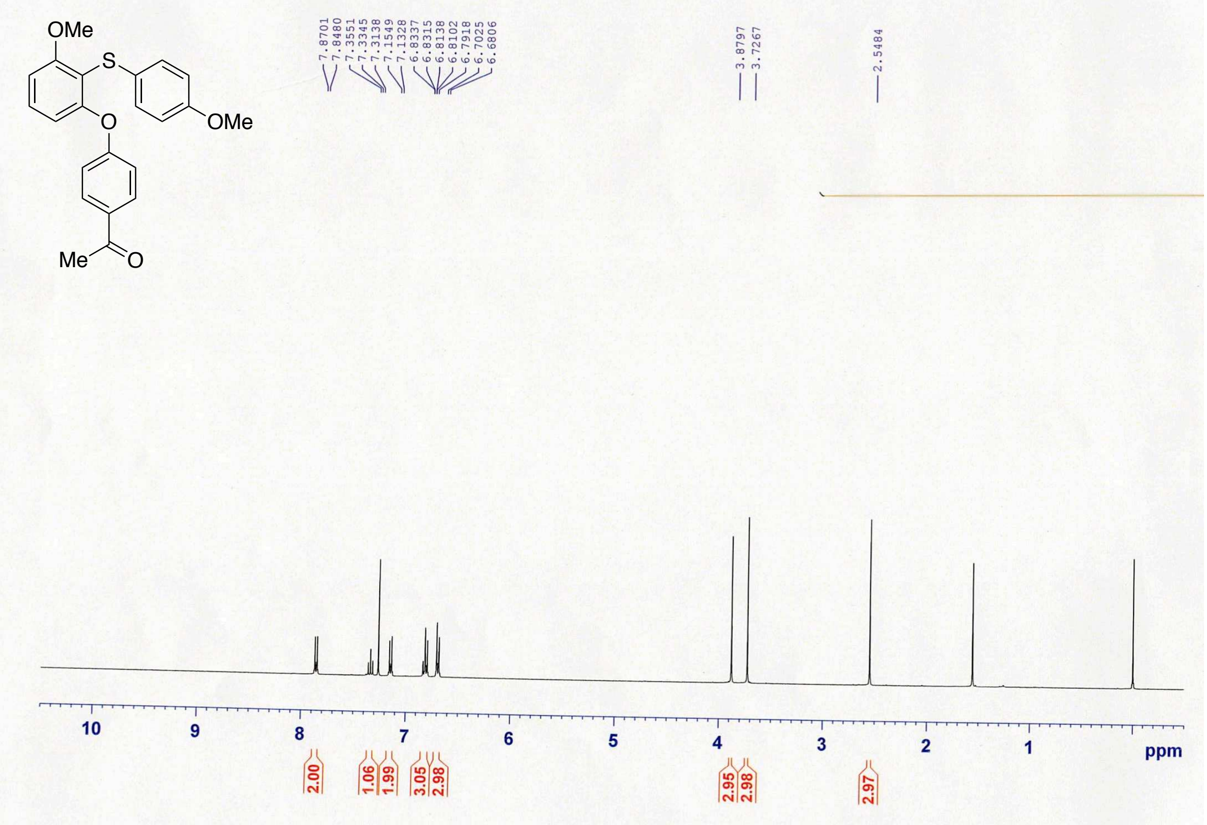

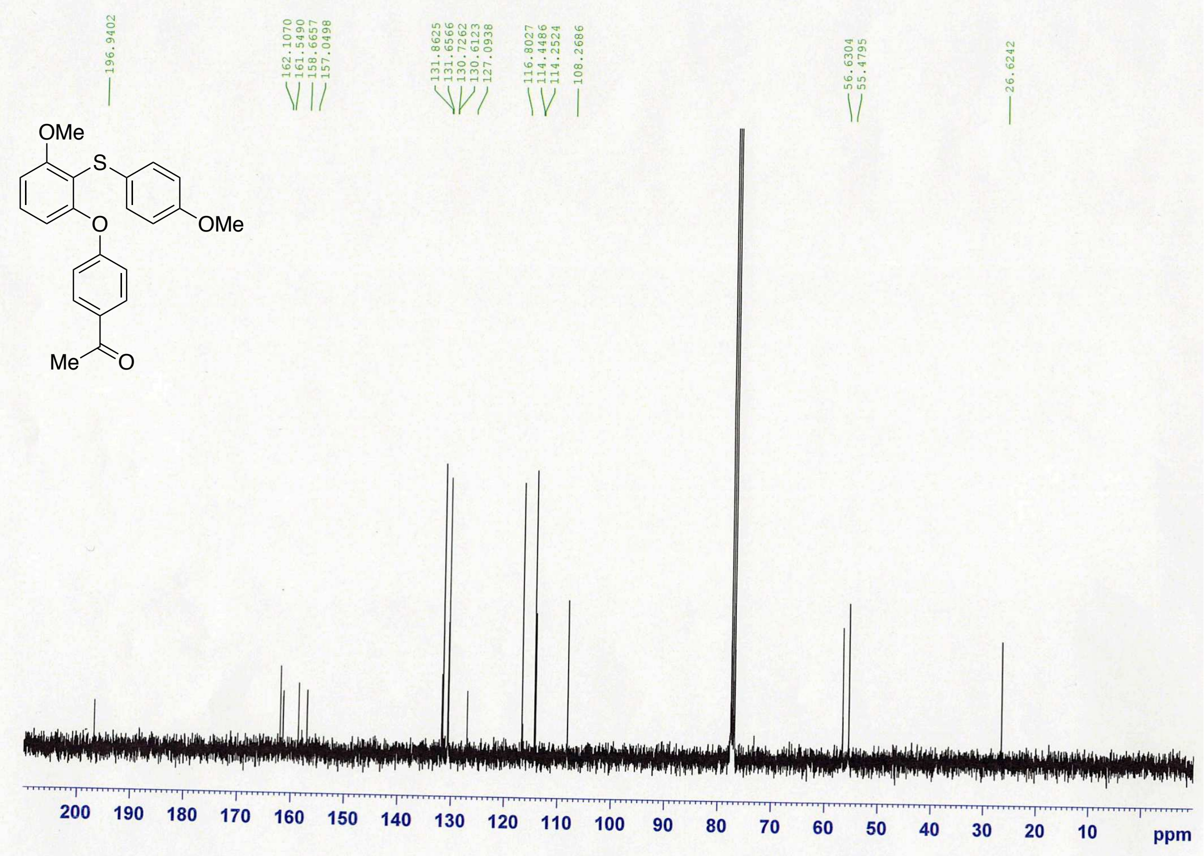

^{1}H NMR (500 MHz) and ^{13}C NMR (126 MHz) spectra of 4-acetylphenyl 2-(4-anisylthio)-3-morpholinophenyl ether (**8b**) ($CDCl_3$)

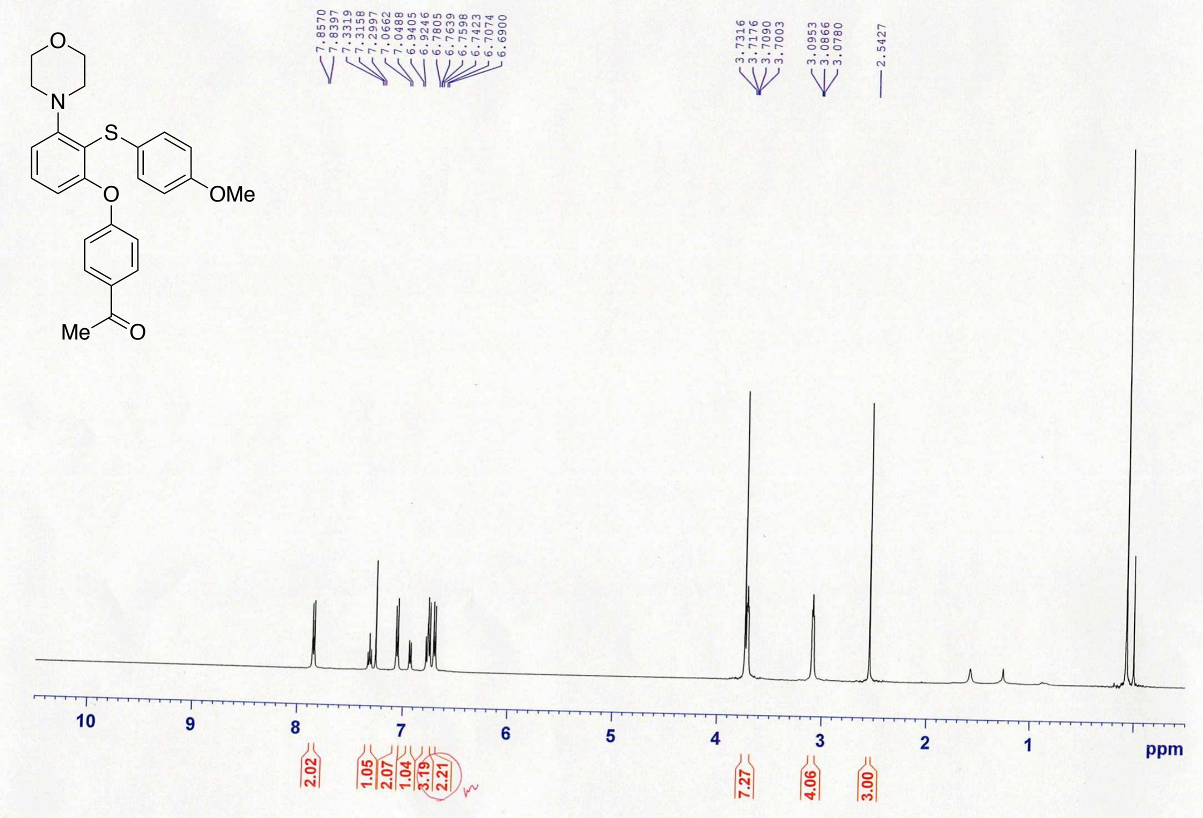

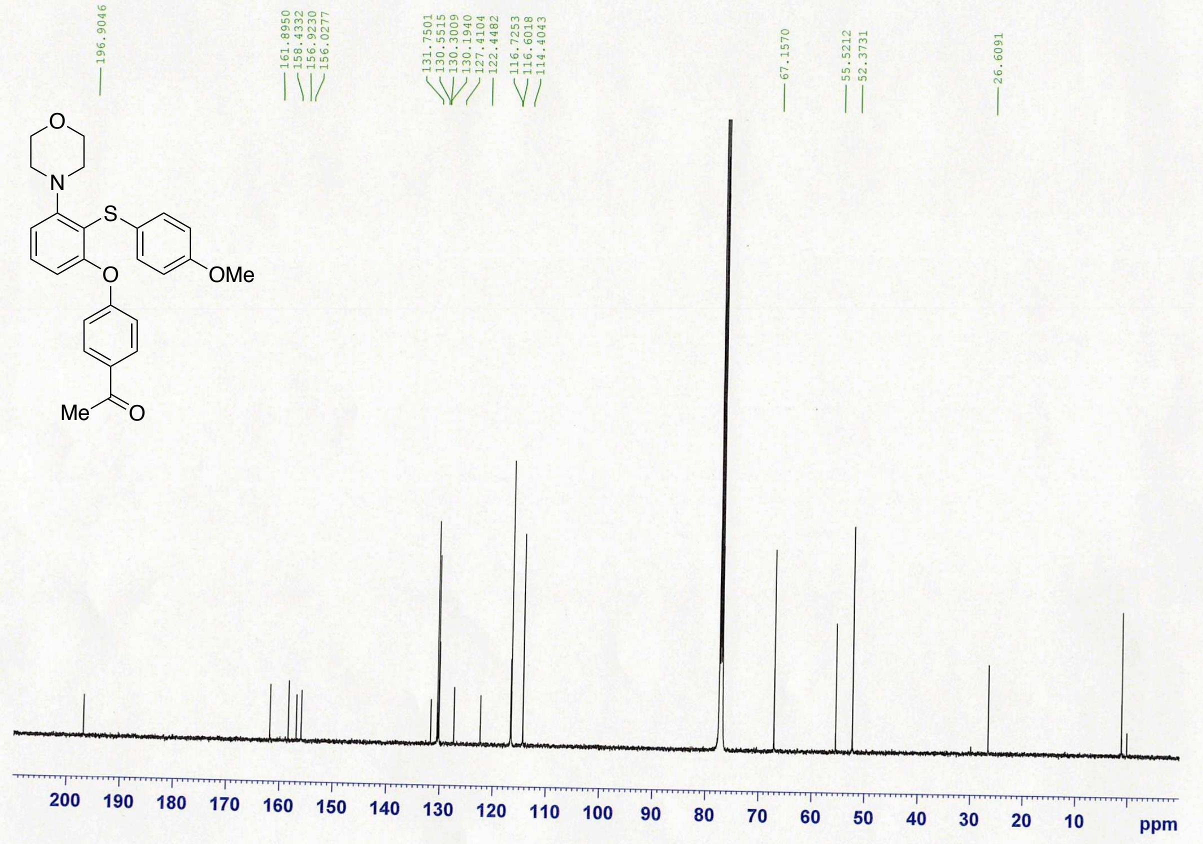

^{1}H NMR (500 MHz) and ^{13}C NMR (126 MHz) spectra of 2-(4-anisylthio)-3-methoxyphenyl *trans*-2-phenylvinyl ether (**8c**) ($CDCl_3$)

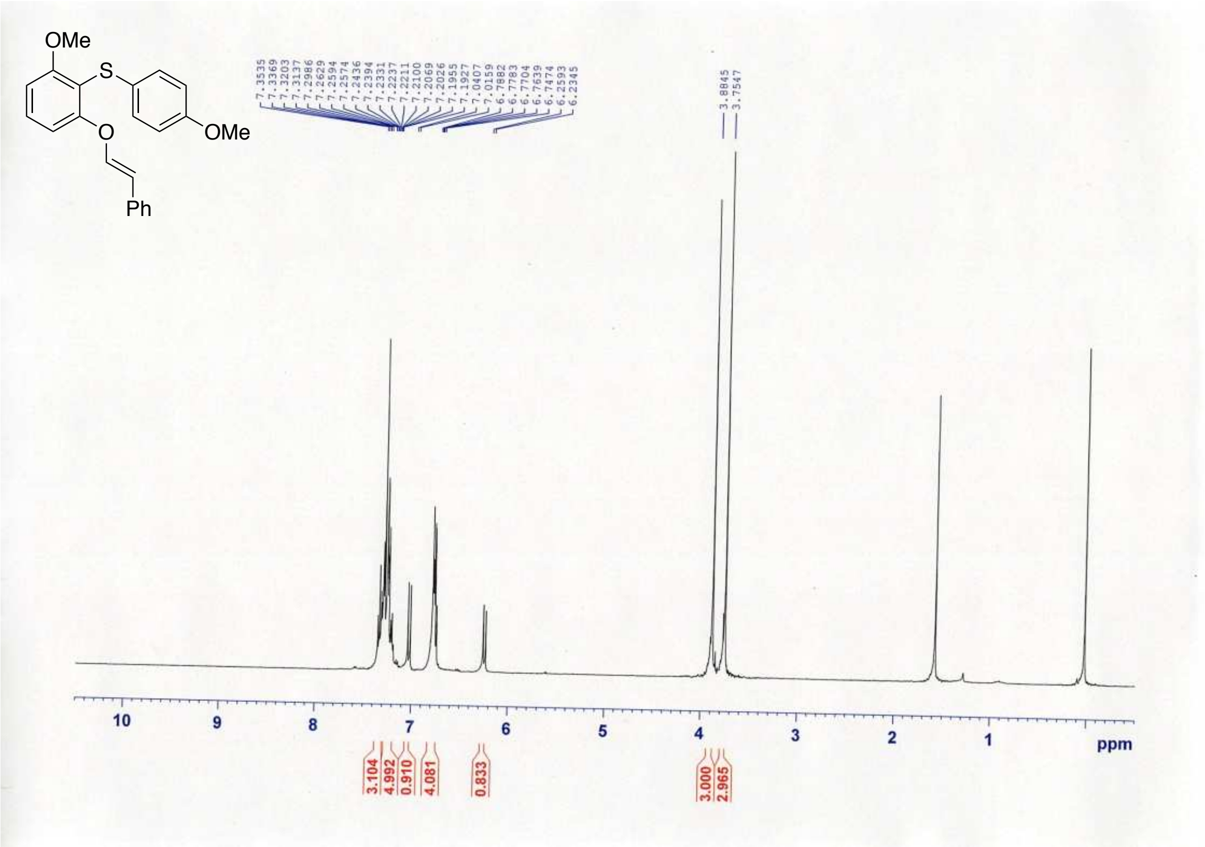

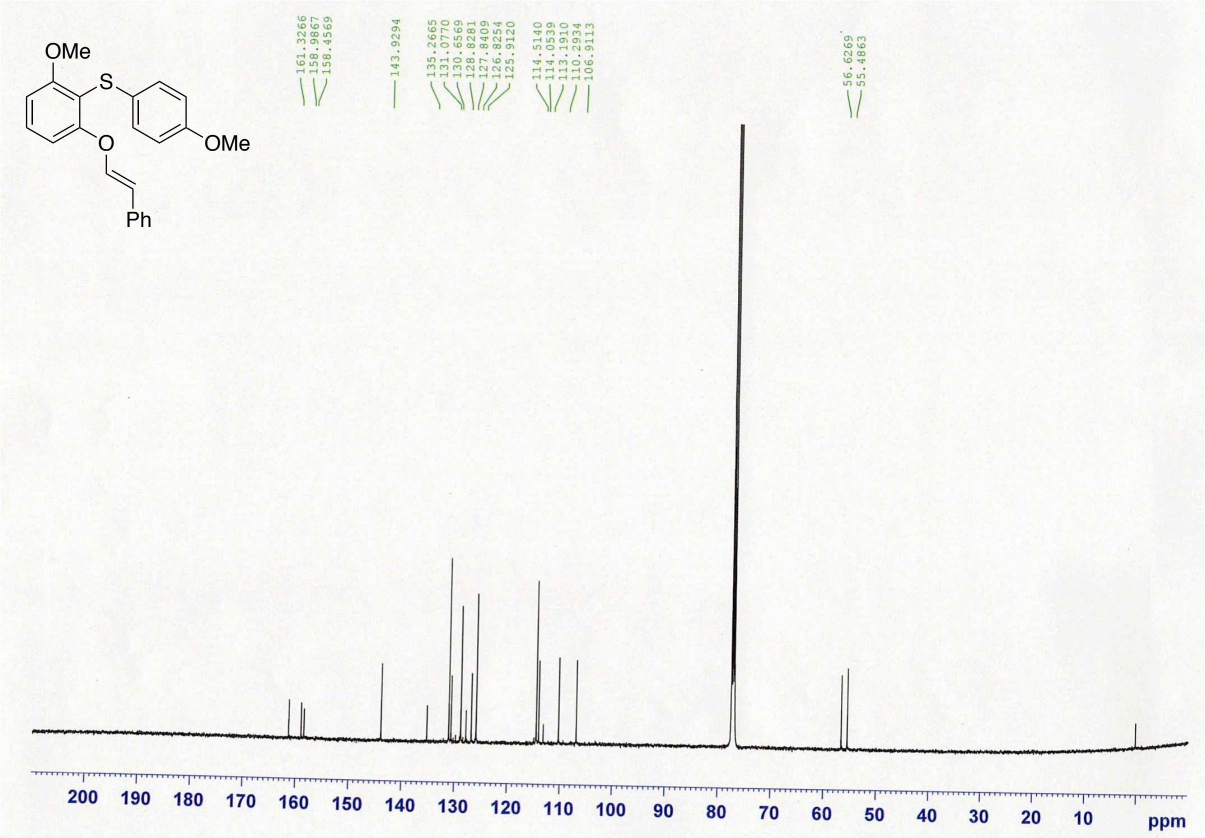

Supplement: Supplementary file 1 — ol1c01292_si_001.pdf [file ol1c01292_si_001.pdf]
